# Hedgehog-Regulated Ubiquitination Controls Smoothened Trafficking and Cell Surface Expression in *Drosophila*

**DOI:** 10.1371/journal.pbio.1001239

**Published:** 2012-01-10

**Authors:** Shuang Li, Yongbin Chen, Qing Shi, Tao Yue, Bing Wang, Jin Jiang

**Affiliations:** 1 Department of Developmental Biology, University of Texas Southwestern Medical Center at Dallas, Dallas, Texas, United States of America; 2 Department of Pharmacology, University of Texas Southwestern Medical Center at Dallas, Dallas, Texas, United States of America; University of Zurich, Switzerland

## Abstract

Hedgehog transduces signal by promoting cell surface expression of the seven-transmembrane protein Smoothened (Smo) in *Drosophila*, but the underlying mechanism remains unknown. Here we demonstrate that Smo is downregulated by ubiquitin-mediated endocytosis and degradation, and that Hh increases Smo cell surface expression by inhibiting its ubiquitination. We find that Smo is ubiquitinated at multiple Lysine residues including those in its autoinhibitory domain (SAID), leading to endocytosis and degradation of Smo by both lysosome- and proteasome-dependent mechanisms. Hh inhibits Smo ubiquitination via PKA/CK1-mediated phosphorylation of SAID, leading to Smo cell surface accumulation. Inactivation of the ubiquitin activating enzyme Uba1 or perturbation of multiple components of the endocytic machinery leads to Smo accumulation and Hh pathway activation. In addition, we find that the non-visual β-arrestin Kurtz (Krz) interacts with Smo and acts in parallel with ubiquitination to downregulate Smo. Finally, we show that Smo ubiquitination is counteracted by the deubiquitinating enzyme UBPY/USP8. Gain and loss of UBPY lead to reciprocal changes in Smo cell surface expression. Taken together, our results suggest that ubiquitination plays a key role in the downregulation of Smo to keep Hh pathway activity off in the absence of the ligand, and that Hh-induced phosphorylation promotes Smo cell surface accumulation by inhibiting its ubiquitination, which contributes to Hh pathway activation.

## Author Summary

The Hedgehog (Hh) family of secreted proteins governs cell growth and patterning in diverse species ranging from *Drosophila* to human. Hh signals across the cell surface membrane by regulating the subcellular location and conformation of a membrane protein called Smoothened (Smo). In *Drosophila*, Smo accumulates on the cell surface in response to Hh, whereas in the absence of Hh it is internalized and degraded. The molecular mechanisms that control this intracellular trafficking and degradation of Smo were unknown, but here we show that Smo is modified by attachment of several molecules of a small protein called ubiquitin, which tags it for internalization and degradation within the cell. Hh inhibits this ubiquitination of Smo by inducing another modification, phosphorylation, of its intracellular tail by two types of protein kinase enzymes. This loss of ubiquitination and gain of phosphorylation causes the accumulation of Smo at the cell surface. What's more, we find that another protein called Kurtz interacts with Smo and acts in parallel with the ubiquitination process to promote internalization of Smo, and that the deubiquitinating enzyme UBPY/USP8 counteracts ubiquitination of Smo to promote its cell surface accumulation. Our study demonstrates that reversible ubiquitination plays a key role in regulating Smo trafficking to and from the cell surface and thus it provides novel insights into the mechanism of Hh signaling from the outside to the inside of the cell.

## Introduction

Hedgehog (Hh) signaling governs cell growth and patterning in species ranging from insects to human [1],[2]. Because of its pivotal role in embryonic development and adult tissue homeostasis, misregulation of Hh signaling activity has been linked to many human disorders including birth defects and cancers [1],[3],[4]. Hh exerts its biological influence through a largely conserved signaling cascade that culminates at the activation of latent transcription factors Cubitus interruptus (Ci)/Gli [1].

The core Hh reception system consists of a 12-transmembrane protein Patched (Ptc) that acts as the Hh receptor and a seven-transmembrane protein Smo that acts as the Hh signal transducer [5],[6]. Hh and Ptc reciprocally regulate the subcellular localization and active state of Smo [7]–[10]. In *Drosophila*, Hh stimulation or loss of Ptc leads to cell surface accumulation of Smo [7],[11]. Increased cell surface expression and activation of Smo are regulated by Hh-induced and PKA/CK1-mediated phosphorylation of Smo carboxyl intracellular tail (C-tail) [12]–[14].

Several observations suggest that Smo cell surface expression is controlled by endocytic trafficking. A transmission electron microscopic study of *Drosophila* imaginal discs indicated that Smo is localized primarily in the lysosome of anterior compartment cells but is enriched on the plasma membrane of posterior compartment cells [15]. In *Drosophila* salivary gland cells, blocking endocytosis promotes Smo cell surface accumulation [11]. Using antibody uptake assay in S2 cells, we have shown that Smo reaches the cell surface but quickly internalizes in the absence of Hh and that Hh stimulation diminishes internalized Smo with a concomitant increase in cell surface Smo [13]. Taken together, these observations suggest that Hh signaling may regulate Smo cell surface expression by blocking its endocytosis and/or promoting its recycling back to the cell surface after internalization.

The mechanisms by which Smo endocytic trafficking and cell surface expression are regulated have remained unknown. Smo intracellular regions lack recognizable endosomal-lysosomal sorting signals such as the NPXY and dileucine-based motifs [16]. However, many membrane receptors are internalized after covalently modified by ubiquitination, as has been demonstrated for receptor tyrosine kinases (RTKs) and G protein coupled receptors (GPCRs) [17],[18]. The close relationship between Smo and GPCRs prompted us to investigate whether Smo cell surface expression is regulated by the ubiquitin pathway. Here we provide both genetic and biochemical evidence that Smo trafficking and degradation are regulated through multi-site ubiquitination of Smo C-tail and that Hh promotes Smo cell surface expression by inhibiting its ubiquitination. We also provide evidence that the non-visual β-arrestin Kurtz (Krz) acts in parallel with Smo ubiquitination to control Smo cell surface expression, and that the deubiquitinating enzyme UBPY promotes Smo cell surface expression by counteracting Smo ubiquitination.

## Results

### Inactivation of the Ubiquitin-Activating Enzyme Uba1 Leads to Smo Accumulation

**Figure 1:**
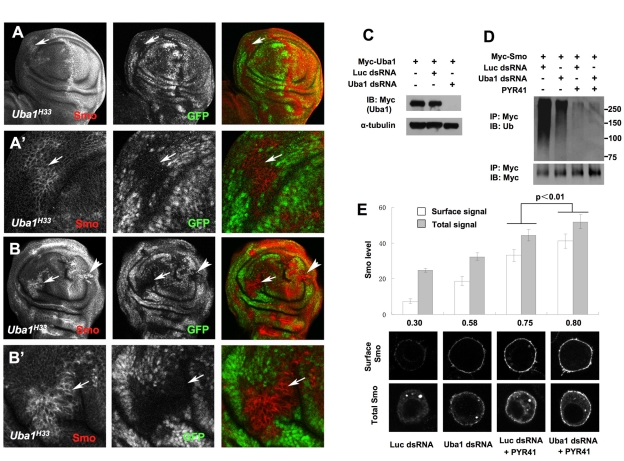
Uba1 regulates Smo ubiquitination and cell surface expression. (A–B') Low (A, B) and high (A', B') magnification view of wing imaginal discs carrying *Uba1^H33^* mutant clones and immunostained with anti-SmoN (red) and anti-GFP (green) antibodies. *Uba1^H33^* mutant clones are marked by the lack of GFP staining. Arrows and arrowheads indicate anterior and posterior clones, respectively. (C) The efficiency of Uba1 RNAi was evaluated by Western blot analysis of transfected Myc-Uba1. (D) S2 cells stably expressing a Myc-tagged Smo under the control of *metallothionein* promoter were treated with Uba1 dsRNA or control (Luciferase) dsRNA in the absence or presence of the E1 inhibitor PYR41. After treatment with MG132, cells extracts were prepared and immunoprecipitated with anti-Myc antibody, followed by Western blot analysis with an anti-Ub antibody to visualize ubiquitinated Smo (top) or anti-Myc antibody to visualize Myc-Smo (bottom). Loading was normalized by the amount of Myc-Smo monomer. IP, immunoprecipitation; IB, immunoblot. (E) S2 cells stably expressing Myc-Smo were treated as in (D). Cells were immunostained with anti-SmoN antibody before membrane permeabilization to visualize cell surface Smo (top panels) or after membrane permeabilization to examine the total Smo (bottom panels). Quantification of cell surface and total Smo levels was shown (20 cells for each condition). The numbers indicate the ratio of cell surface Smo signal versus total Smo signal.

In *Drosophila* wing discs, Smo cell surface level is low in anterior (A) compartment cells away from the A/P boundary but is elevated in response to Hh in A-compartment cells near the A/P boundary or in posterior (P) compartment cells (Figure 1A) [7]. To determine whether Smo is downregulated by the ubiquitin pathway, we generated mutant clones for *Uba1*, which encodes the only ubiquitin-activating enzyme (E1) in *Drosophila* [19],[20]. We employed a temperature-sensitive allele of *Uba1, Uba1^H33^*, which behaves like a null allele at the restrictive temperature [19]. *Uba1^H33^* clones were induced at second instar larval stage (48–72 h AEL) by FRT/FLP mediated mitotic recombination. Larva carrying *Uba1^H33^* clones were grown at permissive temperature (18°C) for 3 d and then shifted to non-permissive temperature (30°C) for 24 h before dissection for immunostaining. We found that anteriorly situated *Uba1^H33^* clones accumulated high levels of Smo compared with neighboring wild type cells (Figure 1A–B'), suggesting that Smo is downregulated via the ubiquitin pathway in the absence of Hh. Immunostaining with anti-Smo antibody before membrane permeabilization suggested that Smo was accumulated on the cell surface in anteriorly situated *Uba1^H33^* clones (Figure S1A–A'). A 12-h temperature shift resulted in a less robust Smo accumulation in *Uba1^H33^* clones (Figure S1B–B'), likely due to the perdurance of Uba1 activity. In general, Smo elevation coincided well with *Uba1* mutant clones. Intriguingly, *Uba1^H33^* mutant cells situated in the posterior compartment also exhibited slightly higher levels of Smo than neighboring wild type cells (arrowhead in Figure 1B), suggesting that a fraction of Smo still undergoes ubiquitin-mediated degradation in the presence of Hh.

### Uba1 Regulates Smo Ubiquitination and Cell Surface Expression

To examine whether Smo is directly ubiquitinated and whether Uba1 is responsible for this activity, we carried out a cell-based ubiquitination assay (see Materials and Methods) [21]. We employed RNAi and/or pharmacological inhibitor to inactivate Uba1. S2 cells stably expressing a Myc-tagged Smo (Myc-Smo) were treated with Uba1 or control double-stranded RNA (dsRNA) in the absence or presence of PYR-41, a cell permeable E1 inhibitor [22]. The efficiency of Uba1 RNAi was confirmed by Western blot analysis of an exogenously expressed tagged Uba1 (Figure 1C). Myc-Smo was ubiquitinated efficiently in the absence of Uba1 inhibition (Figure 1D); however, ubiquitination of Smo was attenuated by Uba1 RNAi and more significantly inhibited by PYR-41 (Figure 1D). The incomplete blockage of Smo ubiquitination by Uba1 RNAi is likely due to partial inactivation of Uba1 by the RNAi approach. Indeed, a combined treatment with Uba1 RNAi and PYR-41 resulted in a more complete inhibition of Smo ubiquitination (Figure 1D).

We next applied a cell-based immunostaining assay to determine whether Uba1 regulates Smo cell surface expression [13]. Myc-Smo expressing cells were treated with control or Uba1 dsRNA in the absence or presence of PYR-41. Cell surface and total Smo were visualized by immunostaining with an anti-SmoN antibody prior to and after cell membrane permeabilization, respectively. As shown in Figure 1E, inhibition of Uba1 either by RNAi or PYR-41 increased the levels of Smo cell surface expression and combined treatment resulted in more dramatic cell surface accumulation of Smo.

### Perturbation of Endocytic Machinery Leads to Smo Accumulation and Hh Pathway Activation

**Figure 2:**
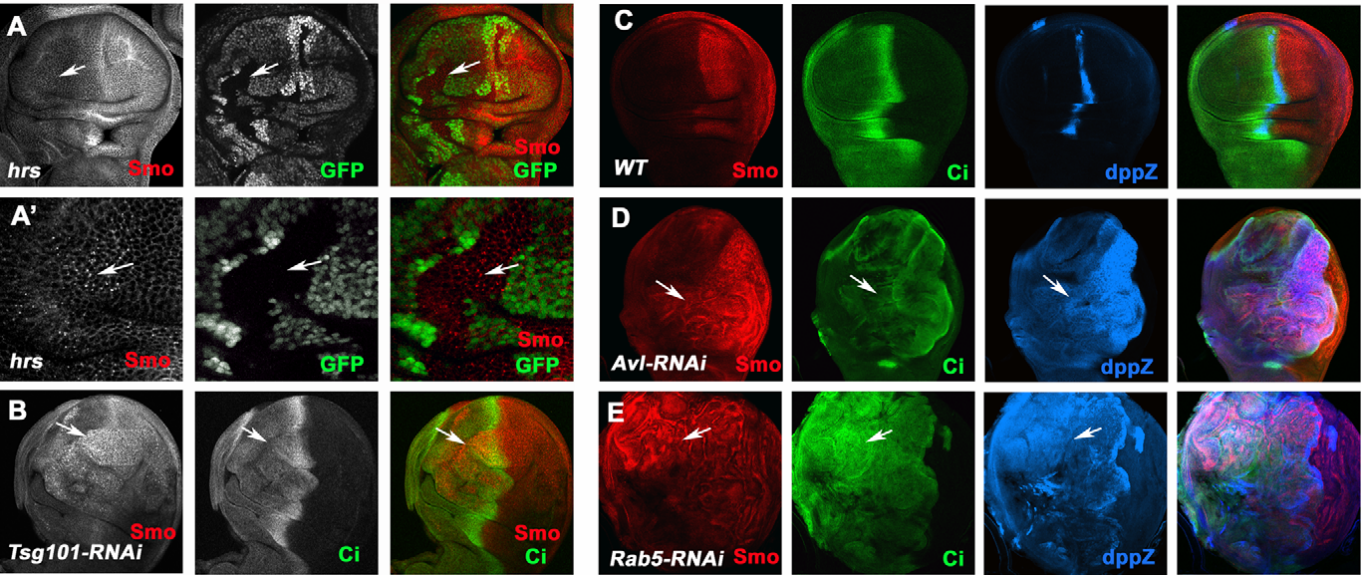
Smo accumulates in cells defective in the endocytic machinery. (A–A') Low (A) and high (A') magnification view of a wing imaginal disc carrying *hrs* mutant clones and immunostained with anti-SmoN (red) and anti-GFP (green) antibodies. *hrs* mutant clones are marked by the lack of GFP staining (arrows). (B–E) A wild type wing disc (C) or wing discs expressing *UAS-Tsg101-RNAi* (B), *UAS-Avl-RNAi* (D), or *UAS-Rab5-RNAi* (E) with the *MS1096* Gal4 driver were immunostained to show the expression of Smo (red), Ci (green), and *dpp-lacZ* (blue). Arrows indicate Smo and Ci accumulation (B, D, E) as well as ectopic *dpp-lacZ* expression (D, E) in A-compartment cells situated distantly from the A/P boundary. Of note, *UAS-Dicer2* was coexpressed with *UAS-Tsg101-RNAi* and *UAS-Avl-RNAi* to enhance the RNAi effect.

Ubiquitinated membrane proteins are internalized through the endocytic pathway and targeted to lysosome for degradation [17]. We therefore examined the effect of inactivation of endocytic components on Smo accumulation in wing imaginal discs. We found that Smo was accumulated in intracellular puncta in mutant clones lacking the *Drosophila* homolog of HGF-regulated tyrosine kinase substrate (Hrs) (Figure 2A–A'), a protein involved in sorting ubiquitinated membrane proteins into multivesicular bodies (MVBs) [23]. Of note, not all *hrs* mutant cells exhibited Smo puncta. This could be due to perdurance of Hrs activity and/or disc folding so that Smo puncta are present at different focal planes. RNAi of other endocytic components, including Tsg101 [24], Avalanche (Avl), a *Drosophila* syntaxin located in early endosomes [25], and Rab5, resulted in Smo accumulation in anterior compartment cells distant from the A/P boundary (arrows in Figure 2B–E), as well as Hh pathway activation as indicated by Ci accumulation and ectopic expression of a Hh target gene *decapentaplegic* (*dpp*) (Figure 2B–E). Taken together, these observations suggest that Smo is downregulated via the endocytic pathway in the absence of Hh.

### Smo Is Degraded by Both Lysosome and Proteasome

**Figure 3:**
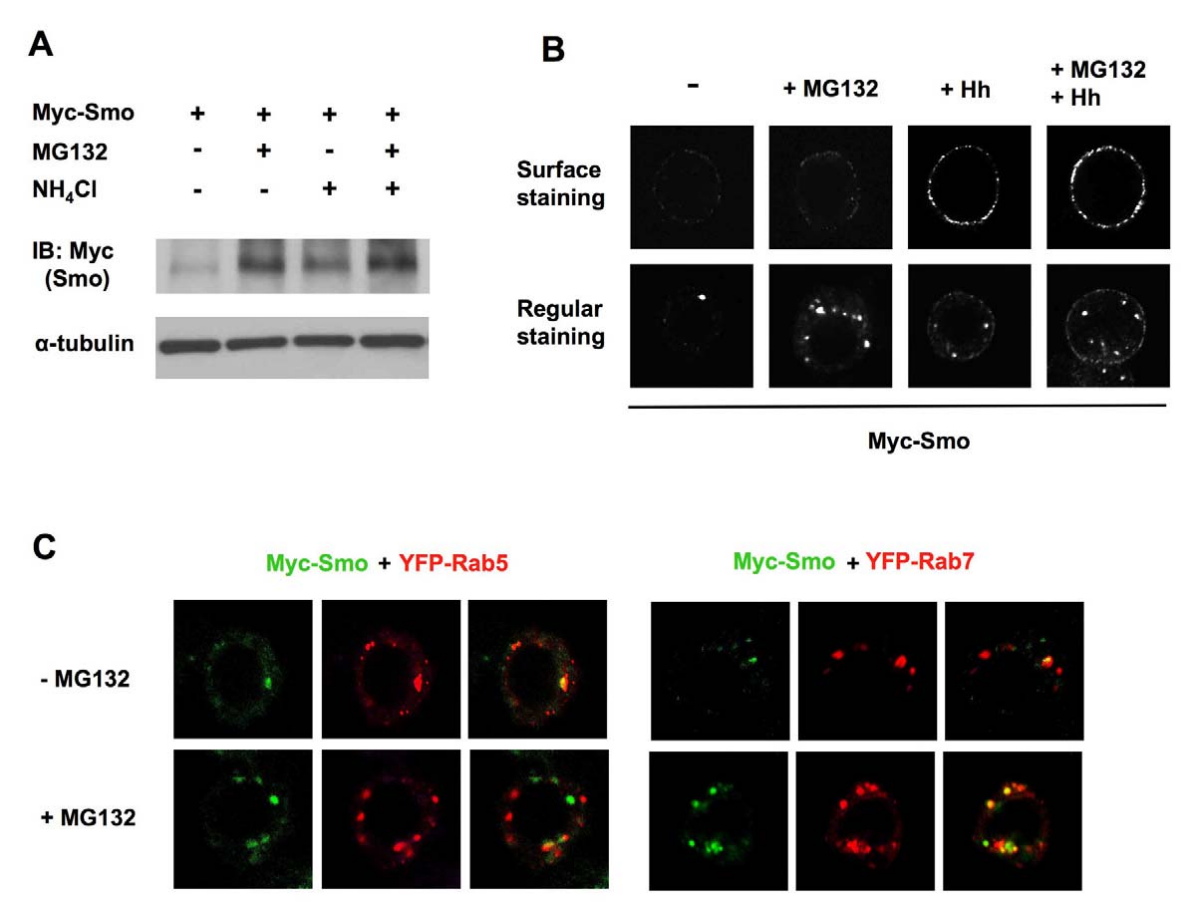
Smo is stabilized by both lysosome and proteasome inhibitors. (A) S2 cells stably expressing Myc-Smo were treated with MG132 and/or NH_4_Cl alone or in combination, followed by Western blot analysis with an anti-Myc antibody. (B) S2 cells stably expressing Myc-Smo treated with or without MG132 and/or Hh-conditioned medium were immunostained with anti-SmoN antibody before membrane permeabilization to visualize cell surface Smo (top panels) or after membrane permeabilization to examine the total Smo (bottom panels). MG132 treatment stabilized Smo in intracellular vesicles whereas Hh treatment led to cell surface accumulation of Smo. (C) Myc-Smo expressing S2 cells were transfected with YFP tagged Rab5 or Rab7, treated with or without MG132 and immunostained to show the expression of Myc-Smo (green) and Rab5/Rab7 (red).

Consistent with Smo being downregulated through the endocytic pathway, treating Myc-Smo expressing S2 cells with a lysosome inhibitor, NH_4_Cl, stabilized Smo (Figure 3A). Interestingly, treating cells with a proteasome inhibitor, MG132, stabilized Smo more dramatically than treating cells with NH_4_Cl (Figure 3A). Furthermore, combined treatment of cells with MG132 and NH_4_Cl had an additive effect on Smo stabilization (Figure 3A), suggesting that Smo is downregulated by both lysosome- and proteasome-dependent mechanisms. However, unlike the case of Hh stimulation or Uba1 inaction where Smo was accumulated on the cell surface, proteasome inhibition stabilized Smo in intracellular vesicles (Figure 3B). Double labeling with endosomal markers YFP-Rab5 (for early endosomes) or YFP-Rab7 (for late endosomes) revealed that Smo was stabilized in Rab7 positive late endosomes after MG132 treatment (Figure 3C). Taken together, these observations suggest that a fraction of internalized Smo was degraded by proteasome in the endocytic pathway before reaching to lysosome.

### Hh Inhibits Smo Ubiquitination Via PKA/CK1-Mediated Phosphorylation

**Figure 4:**
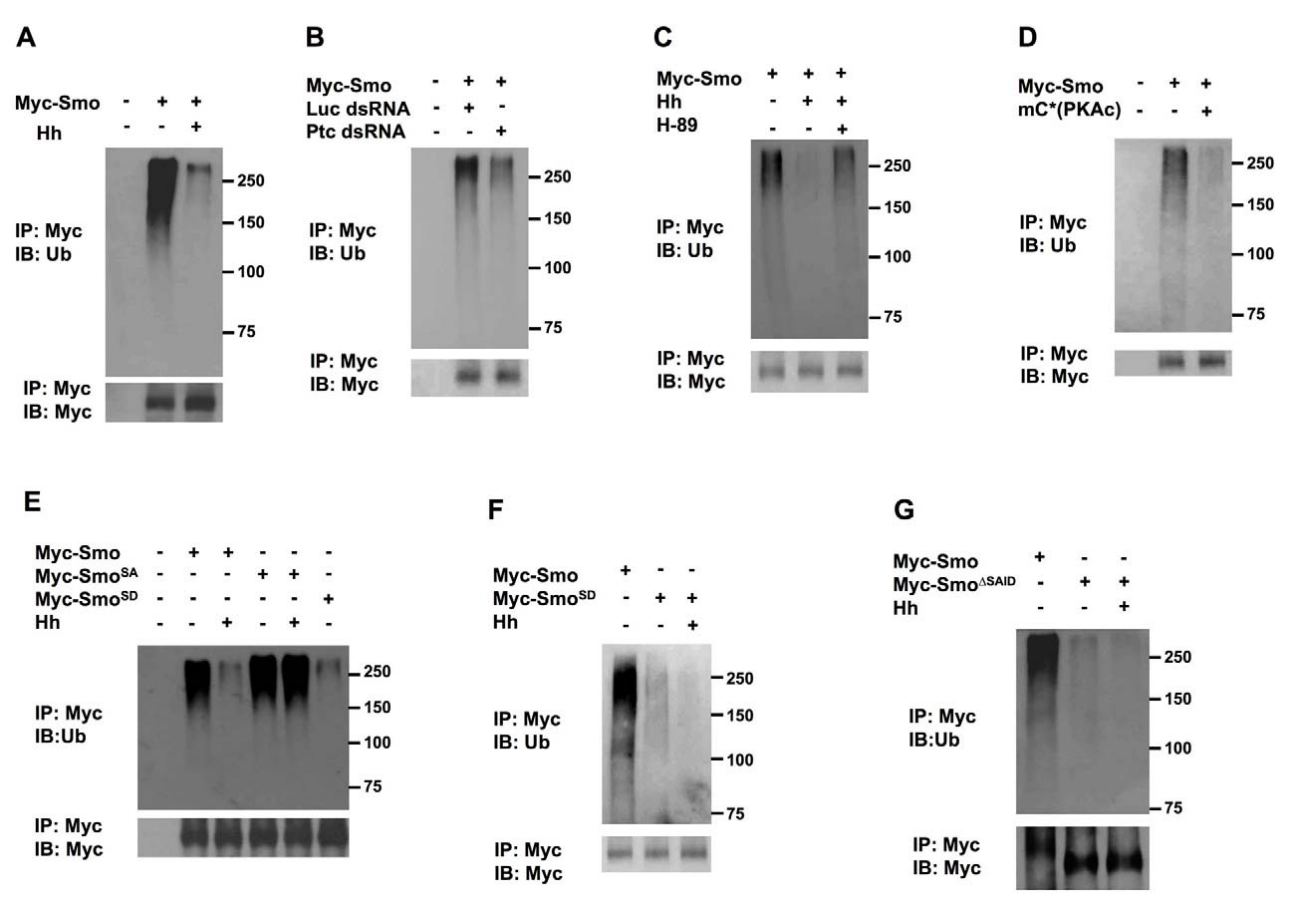
Smo ubiquitination is inhibited by Hh and PKA/CK1-mediated phosphorylation.

Hh induces Smo cell surface accumulation both in vitro and in vivo [7],[11],[13]. If Smo ubiquitination is responsible for its internalization, Hh may increase Smo cell surface expression by inhibiting its ubiquitination. Indeed, treating Myc-Smo stably expressing cells with Hh-conditioned medium markedly reduced but did not completely abolish Smo ubiquitination (Figure 4A). Similarly, Ptc RNAi also reduced Smo ubiquitination (Figure 4B).

Our previous study demonstrated that Hh induced Smo cell surface accumulation through PKA/CK1-mediated phosphorylation of Smo C-tail [13]. We therefore determined whether Hh regulates Smo ubiquitination in a manner depending on Smo phosphorylation. We found that Hh stimulation failed to inhibit Smo ubiquitination in the presence of a PKA inhibitor H-89 (Figure 4C). On the other hand, expressing a constitutively active PKA catalytic domain (mC*) inhibited Smo ubiquitination in the absence of Hh (Figure 4D). To further determine whether Smo ubiquitination is regulated by PKA/CK1-mediated phosphorylation of its C-tail, S2 cells were transfected with Myc-tagged wild type Smo, a phosphorylation deficient form of Smo (Smo^SA^) with three PKA sites (S667, S687, and S740) mutated to Ala, or a phospho-mimetic form of Smo (Smo^SD^) with three PKA/CK1 clusters mutated to Asp [13], treated without or with Hh conditioned medium, and followed by the ubiquitination assay described above. As shown in Figure 4E, Hh inhibited the ubiquitination of Myc-Smo but did not significantly affect the ubiquitination of Myc-Smo^SA^. Furthermore, the phospho-mimetic Smo mutant, Smo^SD^, exhibited diminished ubiquitination and its residual ubiquitination was further reduced by Hh treatment (Figure 4E–F). These results support the notion that Hh-induced phosphorylation by PKA/CK1 inhibits Smo ubiquitination, leading to its cell surface accumulation.

### The SAID Domain Promotes Smo Ubiquitination and Endocytosis

Our previous study revealed that the Smo autoinhibitory domain (SAID) inhibits Smo activity in part by preventing Smo cell surface expression because a Smo variant lacking the SAID domain (Smo^Δ661–818^ or Smo^ΔSAID^) accumulated on the cell surface in the absence of Hh stimulation [8]. To determine whether the SAID domain regulates Smo ubiquitination, we examined the ubiquitin status of a Myc-tagged Smo^ΔSAID^ (Myc-Smo^ΔSAID^). As shown in Figure 4G, deleting the SAID domain diminished Smo ubiquitination, and the residual ubiquitination of Myc-Smo^ΔSAID^ was further reduced by Hh treatment.

**Figure 5:**
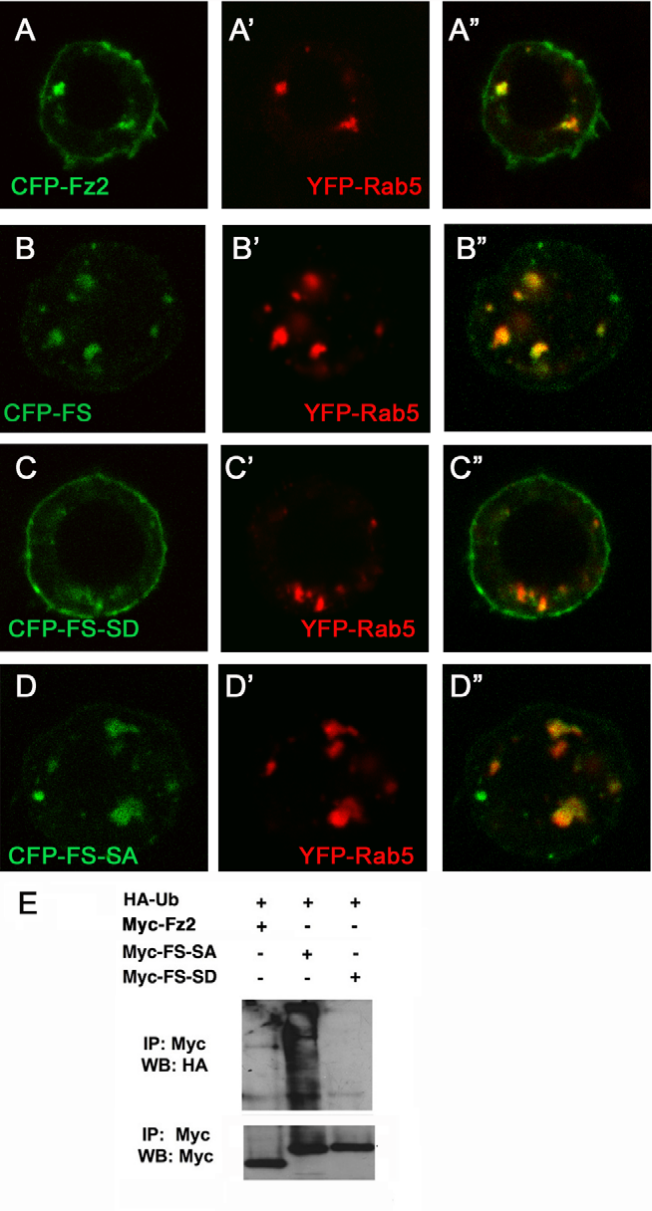
The SAID domain promotes ubiquitination and endocytosis of a heterologous protein. (A–D) Confocal images of S2 cells transfected with CFP-tagged Fz2 (A), Fz2-SAID fusion (FS in B), Fz2-SAID with either the phospho-mimetic (FS-SD in C), or the phosphorylation deficient (FS-SA in D) mutations together with YFP-Rab5. Addition of the wild type or phosphorylation deficient but not the phospho-mimetic form of SAID to Fz2 increased its endocytosis and colocalization with Rab5. (E) Myc-tagged Fz2, FS-SA, and FS-SD were transfected into S2 cells with HA-Ub. Cell lysates were immuno-precipitated (IP) with anti-Myc antibody, followed by Western blot with anti-HA (top panel) and anti-Myc (bottom panel) antibodies.

To determine whether the SAID domain suffices to promote ubiquitination and internalization of a heterologous membrane protein, we fused it to the C-terminus of the Wingless (Wg) receptor Frizzle 2 (Fz2) to construct Fz2-SAID chimeric protein (FS). When expressed in S2 cells, CFP-tagged Fz2 (CFP-Fz2) was largely accumulated on the cell surface with a small fraction internalized and colocalized with the endosomal marker Rab5 (Figure 5A–A”). In contrast, CFP-FS was barely detectable on the cell surface but largely accumulated in Rab5-positive endosomes (Figure 5B–B”), suggesting that SAID can promote endocytosis of Fz2. Introducing the phosphorylation-mimetic mutation to the SAID domain of FS (CFP-FS-SD) reduced its endocytosis (Figure 5C–C”), whereas the chimeric protein carrying a phosphorylation deficient form of SAID (CFP-FS-SA) was internalized as efficiently as CFP-FS (Figure 5D–D”). In addition, we found that adding the phosphorylation-deficient form but not the phospho-mimetic form of SAID to Fz2 promotes the ubiquitination of the corresponding chimeric protein (Figure 5E). Taken together, these observations suggest that the SAID domain suffices to promote ubiquitination and internalization of a membrane protein in a manner inhibited by phosphorylation. Combined with our earlier work [8], it seems that the SAID domain autonomously regulates ubiquitination independent of the C-terminal negatively charged region.

### Smo Is Ubiquitinated at Multiple Lysine Residues

**Figure 6:**
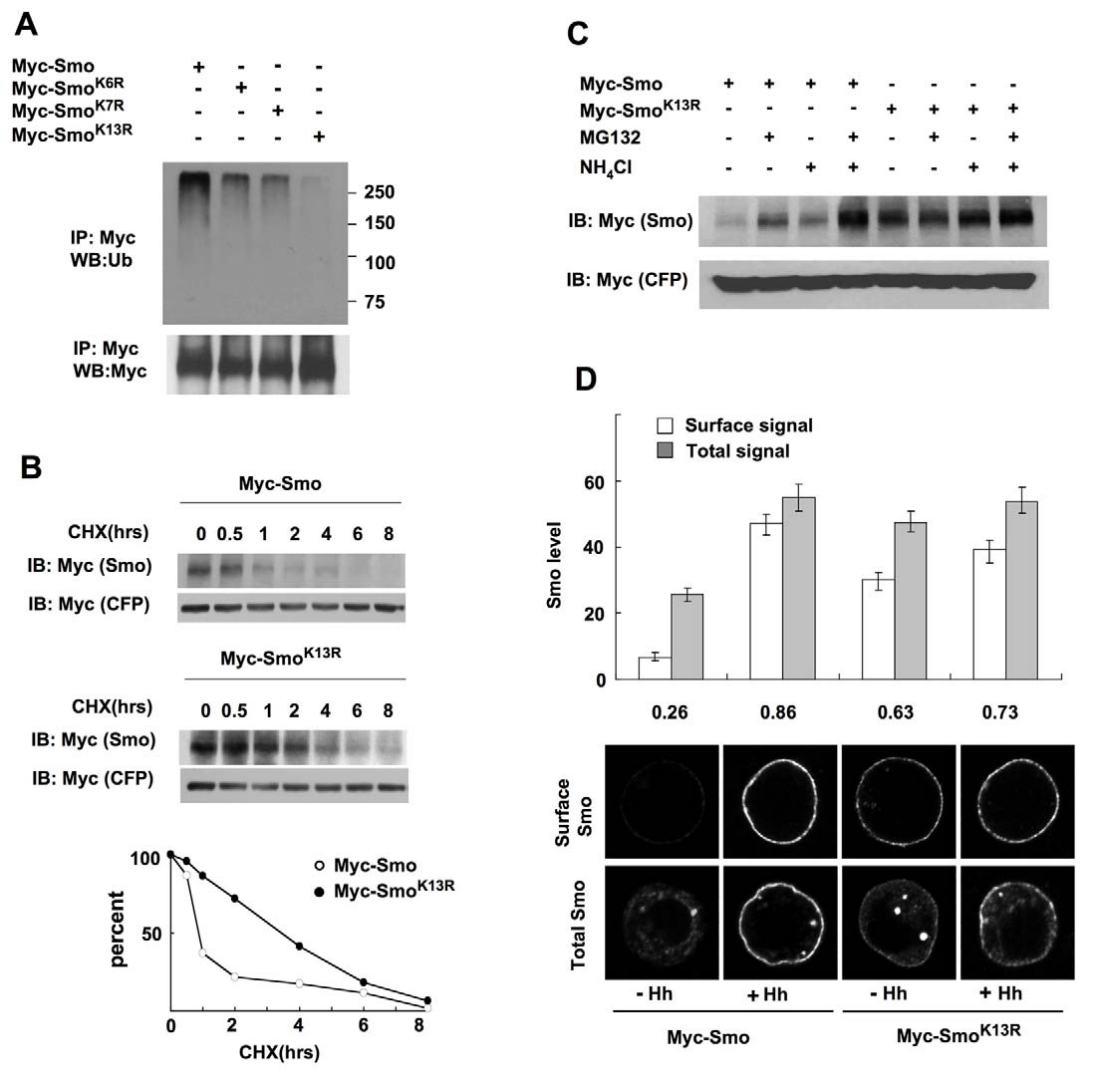
Smo is internalized and degraded by multi-site ubiquitination. (A) Cell extracts from S2 cells transfected with Myc-Smo, Myc-Smo^K6R^, Myc-Smo^K7R^, or Myc-Smo^K13R^ were immunoprecipitated with anti-Myc antibody, followed by Western blot analysis with anti-Ub (top) or anti-Myc antibody (bottom). (B) S2 cells were transfected with Myc-Smo or Myc-Smo^K13R^ together with Myc-CFP (as internal control) and treated with cycloheximide (CHX) for the indicated time. Cell extracts were subjected to Western blot analysis with anti-Myc antibody. Quantification of the Western blot analysis is shown at bottom. (C) S2 cells were transfected with Myc-Smo or Myc-Smo^K13R^ together with Myc-CFP and treated without or with MG132 and/or NH_4_Cl. Cell extracts were subjected to Western blot analysis with anti-Myc antibody. (D) S2 cells transfected with Myc-Smo or Myc-Smo^K13R^ and treated with or without Hh-conditioned medium were immunostained with anti-SmoN antibody prior to (top panels) or after (bottom panels) membrane permeabilization. Quantification of cell surface and total Smo levels was shown (20 cells for each condition). The numbers indicate the ratio of cell surface Smo signal versus total Smo signal.

If Smo ubiquitination is responsible for its internalization and degradation, one would expect that ubiquitination-deficient Smo variants should be stabilized and accumulated on the cell surface. We therefore attempted to identify Lys residues responsible for Smo ubiquitination. In general, ubiquitin acceptor sites lack a strict consensus and target proteins can be ubiquitinated at multiple Lys residues. Smo C-tail and intracellular loops contain a total of 49 Lys residues, many of which may serve as ubiquitin acceptor sites, making it difficult to generate Smo variants devoid of ubiquitination. As deleting the SAID domain diminished Smo ubiquitination (Figure 4G), we speculated that this region might contain Lys residues critical for Smo ubiquitination. There are a total of 13 Lys residues between aa 661 and aa 818. We therefore constructed Smo^K6R^ with K665, K695, K700, K702, K710, and K733 mutated to Arg; Smo^K7R^ with K752, K753, K762, K772, K773, K782, and K801 mutated to Arg; and Smo^K13R^ with all the 13 Lys residues mutated to Arg. Using the cell-based ubiquitination assay described above, we found that both Myc-Smo^K6R^ and Myc-Smo^K7R^ exhibited reduced ubiquitination compared with Myc-Smo (Figure 6A). The combined mutations (K13R) resulted in a more dramatic reduction in Smo ubiquitination (Figure 6A), suggesting that Smo is ubiquitinated at multiple Lys residues between aa 661 and aa 818. In addition, the residual ubiquitination of Myc-Smo^K13R^ suggests that Smo is also ubiquitinated at one or more Lys residues outside the SAID domain.

### Smo^K13R^ Exhibits Increased Stability and Cell Surface Expression

We next determined whether the K13R mutation affects Smo stability and cell surface expression. Myc-Smo and Myc-Smo^K13R^ expression constructs were transfected into S2 cells together with a Myc-CFP expression construct as an internal control. The levels of Myc-Smo and Myc-Smo^K13R^ were monitored at different time points after treatment with the protein synthesis inhibitor, cycloheximide (CHX). As shown in Figure 6B, Myc-Smo^K13R^ exhibited increased half-life compared with Myc-Smo, suggesting that inhibition of Smo ubiquitination leads to its stabilization. We also measured the steady state levels of Myc-Smo and Myc-Smo^K13R^ in the absence or presence of MG132 and/or NH_4_Cl. While Myc-Smo was stabilized by both MG132 and NH_4_Cl, Myc-Smo^K13R^ was stabilized by NH_4_Cl but insensitive to MG132 treatment (Figure 6C), suggesting that inhibition of Smo ubiquitination blocks its degradation by proteasome.

To determine whether inhibition of Smo ubiquitination leads to its cell surface accumulation, S2 cells were transfected with Myc-Smo or Myc-Smo^K13R^ expression construct, followed by treatment with or without Hh-conditioned medium. Cell surface and total Smo were monitored by immunostaining with the anti-SmoN antibody before and after cell permeabilization, respectively. As shown in Figure 6D, Myc-Smo^K13R^ exhibited higher basal level of cell surface expression than Myc-Smo; however, the level of cell surface Myc-Smo^K13R^ in the absence of Hh was still lower than that of Myc-Smo or Myc-Smo^K13R^ in the presence of Hh (Figure 6D). Thus, although Smo^K13R^ exhibits increased stability and cell surface expression, it is still internalized and degraded by lysosome and can be further stabilized by Hh.

To determine whether the K13R mutation affects Smo stability in vivo, we generated transgenic flies expressing either *UAS-Myc-Smo* or *UAS-Myc-Smo^K13R^* from the same genetic locus using the *phiC31* integration system to ensure similar expression level from different constructs [26]. We used the wing specific Gal4 driver *MS1096* coupled with *tub-Gal80^ts^* to drive a pulse of *UAS-Myc-Smo* or *UAS-Myc-Smo^K13R^* expression by shifting late third instar larvae to the non-permissive temperature for 12 h. After chasing for different periods of time, wing discs were immunostained with an anti-Myc antibody. As shown in Figure S2, after a 10 h chase, Myc-Smo was barely detectable in A-compartment cells distant from the A/P boundary, whereas Myc-Smo^K13R^ persisted in these cells, suggesting that Myc-SmoK^13R^ has a longer half-life than Myc-Smo.

### Krz Promotes Smo Internalization by Binding to Its C-Tail

**Figure 7:**
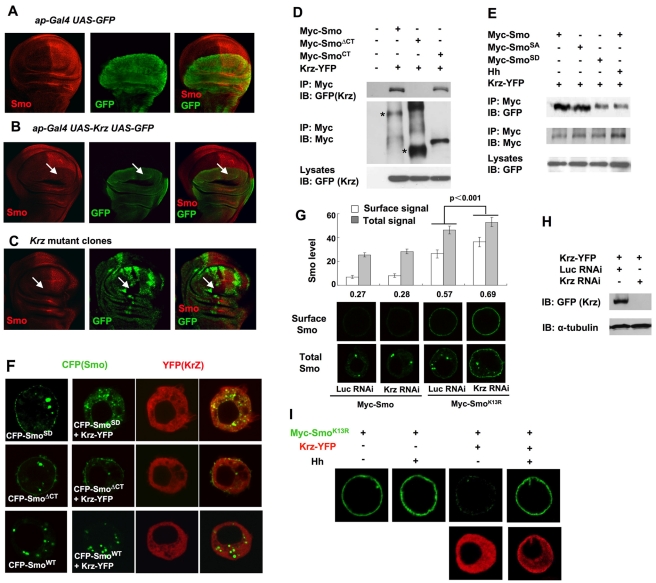
Krz interacts with Smo and downregulates its cell surface expression. (A–B) A wing disc expressing *UAS-GFP* alone (A) or together with *UAS-Krz* (B) under the control of *ap-Gal4* was immunostained with anti-SmoN (red) and anti-GFP (green) antibodies. Krz overexpression cells are marked by GFP in (B). Excessive Krz blocked Smo accumulation in P-compartment cells (arrows in B). (C) A wing imaginal disc carrying *krz* mutant clones was immunostained with anti-SmoN (red) and anti-GFP (green) antibodies. *krz* mutant clones are marked by the lack of GFP staining. Anteriorly situated *krz* mutant clones did not accumulate Smo (arrows). (D–E) S2 cells were transfected with Krz-YFP and Myc-tagged wild type Smo or the indicated Smo variants and treated with or without Hh-conditioned medium. Western blot analyses were carried out on cell lysates or immunoprecipitates using the indicated antibodies. Asterisks indicate monomeric forms of Myc-Smo and Myc-Smo^ΔCT^. (F) Confocal images of S2 cells transfected with CFP-Smo^SD^, CFP-Smo^ΔCT^, or CFP-Smo^WT^ either alone (left) or together with Krz-YFP (right). Overexpression of Krz-YFP internalized CFP-Smo^SD^ but not CFP-Smo^ΔCT^. (G) S2 cells transfected with Myc-Smo or Myc-Smo^K13R^ in the presence of Krz RNAi or Luc RNAi were immunostained with anti-SmoN antibody prior to (top panels) or after (bottom panels) membrane permeabilization. Quantification of cell surface and total Smo levels was shown (20 cells for each condition). The numbers indicate the ratio of cell surface Smo signal versus total Smo signal. (H) Krz RNAi efficiency was evaluated by Western blot analysis of transfected Krz-YFP. (I) S2 cells were transfected with Myc-Smo^K13R^ alone or together with Krz-YFP with or without Hh treatment, followed by immunostaining to visualize cell surface Myc-Smo^K13R^ (green) and Krz-YFP (red).

Internalization of Smo^K13R^ is likely due to its residual ubiquitination at a Lys residue(s) outside the SAID domain. In addition, Smo^K13R^ could also be internalized by Smo interacting proteins, as have been shown for other receptors [27],[28]. It has been shown that the non-visual arrestin, β-arrestin 2, can bind and internalize mammalian Smo [29]. The *Drosophila* non-visual arrestin is encoded by *krz* [30]. We therefore carried out both gain- and loss-of-function studies to determine whether Krz regulates Smo cell surface expression. We found that overexpression of Krz in wing imaginal discs using a dorsal compartment specific Gal4 driver, *ap-Gal4*, blocked Smo accumulation in posterior-dorsal compartment cells (compare Figure 7B with Figure 7A). However, we found that Smo was not accumulated in *krz* mutant clones located in the anterior compartment of wing discs (Figure 7C). Similar observations were obtained by a recent study [31].

Using a coimmunoprecipitation assay, we found that Smo interacted with Krz through its C-tail as both Myc-Smo and Myc-Smo^CT^ (a Smo variant only containing its C-tail) but not Myc-Smo^ΔCT^ (a Smo variant with its C-tail deleted) pulled down a C-terminally YFP-tagged Krz (Krz-YFP) when expressed in S2 cells (Figure 7D). Furthermore, Krz-YFP could internalize Smo^SD^ but not Smo^ΔCT^ in S2 cells (Figure 7F), suggesting that Krz internalizes Smo by binding to its C-tail. The association between Smo and Krz was attenuated by Hh stimulation because Myc-Smo pulled down less Krz-YFP in the presence of Hh conditioned medium (Figure 7E). In addition, Myc-Smo^SD^ pulled down less Krz-YFP than Myc-Smo^SA^ (Figure 7E), suggesting that Smo/Krz interaction is inhibited by Hh and PKA/CK1-mediated phosphorylation.

The observations that overexpression of Krz promoted Smo internalization but its loss of function did not lead to Smo cell surface accumulation suggest that a redundant mechanism(s) may act in parallel with Krz to internalize Smo. For example, in the absence of Krz, ubiquitination of Smo might be sufficient to promote its internalization and degradation. On the other hand, Krz could internalize Smo when Smo ubiquitination is compromised. This may explain, at least in part, why Smo^K13R^ is still internalized and degraded by lysosome. To test this model, we examined the effect of Krz inactivation on the cell surface expression of Myc-Smo and Myc-Smo^K13R^ in S2 cells. Consistent with the finding that loss-of-Krz has no effect on the cell surface expression of endogenous Smo in wing discs (Figure 7C), Krz RNAi did not significantly affect the cell surface expression of Myc-Smo in S2 cells (Figure 7G). In contrast, Krz RNAi increased the cell surface expression of Myc-Smo^K13R^ (Figure 7G), suggesting that Smo^K13R^ is, at least in part, internalized by Krz. Similarly, Krz RNAi enhanced the cell surface accumulation of Myc-Smo induced by Uba1 RNAi or PYR41 (Figure S3), suggesting that Krz acts in parallel with ubiquitination to internalize Smo. On the other hand, overexpression of Krz-YFP blocked the cell surface accumulation of Myc-Smo^K13R^ and this blockage was alleviated by Hh treatment (Figure 7I), suggesting that Hh inhibits Krz-mediated Smo internalization.

### Smo Ubiquitination Is Counteracted by the Deubiquitinating Enzyme UBPY

**Figure 8:**
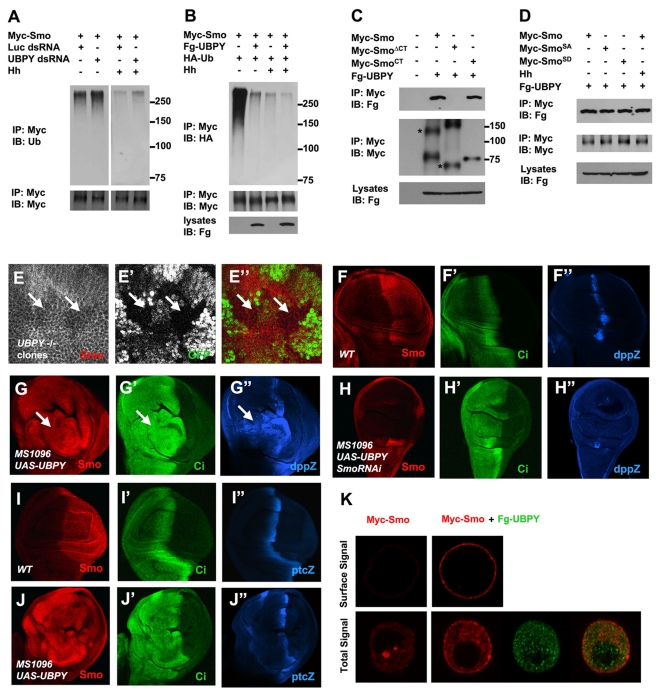
UBPY regulates Smo ubiquitination and cell surface expression. (A) Myc-Smo expressing cells were treated with or without Hh-conditioned medium in the presence of UBPY or Luc dsRNA. After treatment with MG132, cell extracts were prepared and immunoprecipitated with anti-Myc antibody, followed by Western blot analysis with anti-Ub or anti-Myc antibody. Of note, shorter exposure was used for Western blot analysis of samples derived from cells not treated with Hh (left). (B) S2 cells were transfected with Myc-Smo and HA-tagged Ub (HA-Ub) and with or without Flag-tagged UBPY (Fg-UBPY). After treatment with MG132, cell extracts were prepared and immunoprecipitated with anti-Myc antibody, followed by Western blot analysis with anti-HA or anti-Myc antibody. (C–D) S2 cells were transfected with Fg-UBPY and Myc-tagged wild type Smo or the indicated Smo variants and treated with or without Hh-conditioned medium. Western blot analyses were carried out on cell lysates or immunoprecipitates using the indicated antibodies. Asterisks indicate monomeric forms of Myc-Smo and Myc-Smo^ΔCT^. (E–E”) Large magnification view of a wing disc carrying *UBPY* mutant clones and immunostained to show the expression of Smo (red channel) and GFP (green channel). *UBPY* mutant clones are marked by the lack of GFP expression. Posterior *UBPY* mutant clones had reduced cell surface accumulation of Smo (arrows). (F–J”) Wild type wing discs (F–F”, I–I”) or wing discs expressing *UAS-UBPY* alone (G–G”, J–J”) or together with *UAS-Smo-RNAi* (H–H”) under the control of *MS1096* were immunostained to show the expression of Smo (red), Ci (green), and *dpp-lacZ* or *ptc-lacZ* (blue). (K) Confocal images of S2 cells expressing Myc-Smo (red) alone or together with Fg-UBPY (green). Top panels show cell surface staining while bottom panels show regular staining.

Ubiquitination is a reversible process and ubiquitin attached to target proteins can be removed by deubiquitinating enzymes/DUBs [32]. Compared with the large number of E3 ubiquitin ligases that catalyze ubiquitination of targeted proteins, each genome encodes a much smaller number of DUBs. For example, the *Drosophila* genome encodes over 200 annotated E3s but less than 30 annotated DUBs (Flybase; Table S1). To determine whether Smo ubiquitination is regulated by DUBs, we systematically knocked down individual DUBs by RNAi and examined the effect on Smo ubiquitination in S2 cells stably expressing Myc-Smo. From this screen, we found that RNAi of the *Drosophila* UBPY/USP8 significantly increased the basal levels of Smo ubiquitination (Figure S4). The effect of UBPY RNAi on Smo ubiquitination was confirmed by an independent dsRNA for UBPY (Figure 8A). We also found that inactivation of UBPY by RNAi increased Smo ubiquitination in the presence of Hh (Figure 8A), suggesting that UBPY counteracts Smo ubiquitination in both Hh signaling “off” and “on” states. Consistent with UBPY being able to counteract Smo ubiquitination independent of Hh signaling states, overexpression of UBPY reduced Smo ubiquitination in S2 cells both in the absence and presence of Hh (Figure 8B).

We then carried out coimmunoprecipitation assays to determine whether UBPY physically interacts with Smo. As shown in Figure 8C, Myc-Smo and Myc-Smo^CT^ but not Myc-Smo^ΔCT^ pulled down a flag-tagged UBPY (Fg-UBPY) when expressed in S2 cells, suggesting that UBPY interacts with Smo through its C-tail. The association between UBPY and Myc-Smo was not significantly affected by Hh stimulation (Figure 8D). Furthermore, UBPY appears to interact equally well with Myc-Smo, Myc-Smo^SA^, and Myc-Smo^SD^, suggesting that the bulk of Smo/UBPY association is not regulated by Hh signaling.

We next examined the effect of loss- or gain-of-UBPY on Smo cell surface expression. In wing discs carrying *UBPY* mutant clones, Smo cell surface accumulation was attenuated in P-compartment situated UBPY mutant cells (Figure 8E–E”). On the contrary, expression of *UAS-UBPY* using the wing specific Gal4 driver *MS1096* resulted in Smo accumulation in anterior compartment cells away from the A/P boundary (Figure 8G,J). Similarly, overexpression of UBPY in S2 cells markedly increased the cell surface expression of Myc-Smo (Figure 8K). Overexpression of UBPY in wing discs stabilized full-length Ci (Figure 8G',J') and induced ectopic expression of *dpp-lacZ* in anterior dorsal compartment cells where *MS1096* was expressed at high levels (Figure 8G”). Smo RNAi suppressed the ectopic *dpp-lacZ* expression induced by UBPY overexpression as well as the endogenous *dpp-lacZ* expression near the A/P boundary (Figure 8H–H”). However, overexpression of UBPY induced little if any ectopic expression of *ptc-lacZ* (Figure 8J”), which is normally induced by higher levels of Hh signaling than *dpp-lacZ*. Taken together, these results suggest that UBPY can reverse Smo ubiquitination to promote its cell surface accumulation and induce low but not high levels of Hh pathway activation. This is in line with our previous finding that overexpression of wild type Smo only induced low levels of Hh pathway activation and full activation of Smo requires additional steps, including a phosphorylation-mediated conformational switch in Smo C-tail [7]–[10],[13].

### Smo Is Regulated by Both Mono- and Polyubiquitination

**Figure 9:**
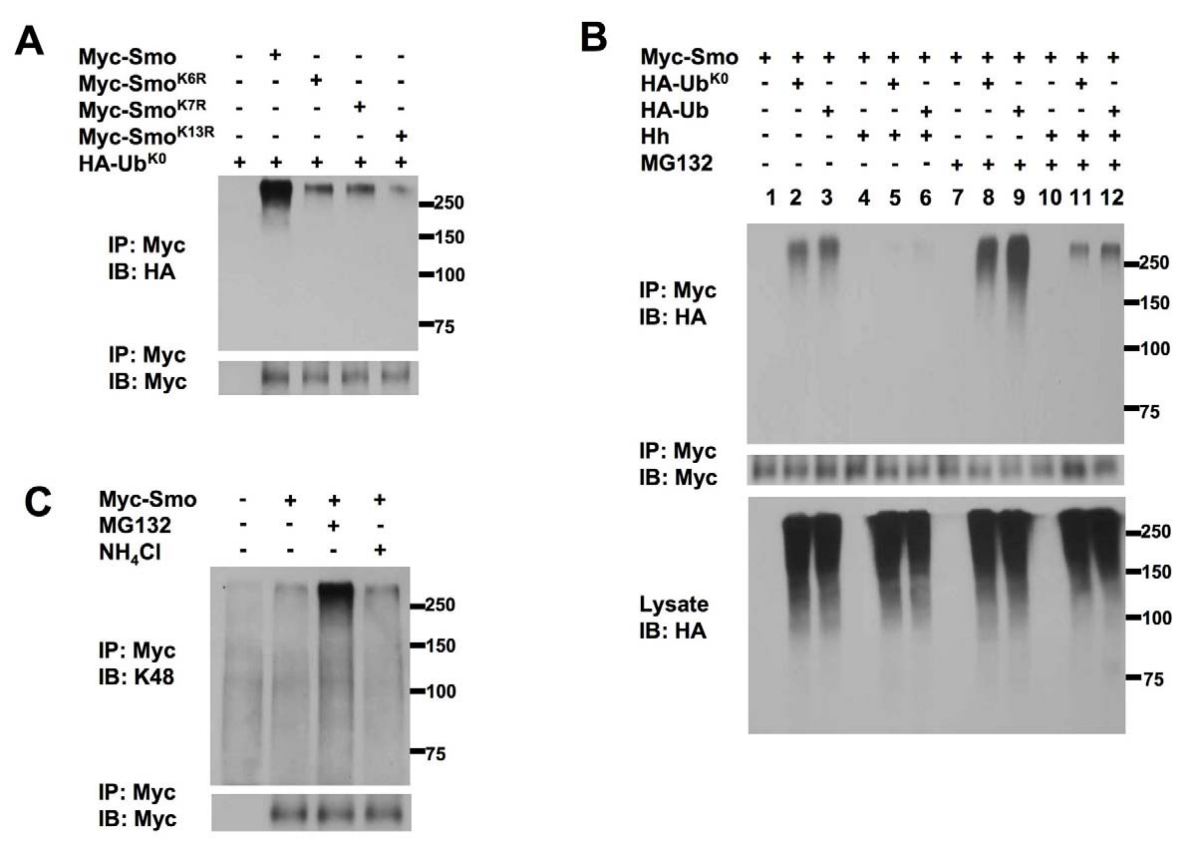
Smo is regulated by both multi- and polyubiquitination. (A) S2 cells were transfected with HA-Ub^K0^ and Myc-Smo or indicated KR variants and treated with NH_4_Cl. Cell extracts were immunoprecipitated with anti-Myc antibody, followed by immunoblotting with anti-Myc and anti-HA antibodies. (B) S2 cells were transfected with Myc-Smo and HA-Ub^K0^ or HA-Ub and treated with or without Hh-conditioned medium and/or MG132. Cell extracts were immunoprecipitated with anti-Myc antibody, followed by immunoblotting with anti-Myc and anti-HA antibodies. The cell lysates were also immunoblotted with anti-HA antibody. (C) Myc-Smo expressing S2 cells or control cells were mock treated, or treated with either MG132 or NH_4_Cl. Cell extracts were immunoprecipitated with anti-Myc antibody, followed by immunoblotting with anti-Myc antibody or a Lys 48-linkage specific polyubiquitin antibody (K48). Of note, Loading was normalized by the amount of Myc-Smo monomer.

It is generally thought that monoubiquitination or multiubiquitination (monoubiquitination at multiple sites) is responsible for receptor internalization and degradation by lysosome, whereas Lys 48-linked polyubiquitination targets proteins for proteasome-mediated degradation. The observation that Smo is degraded by both lysosome and proteasome dependent mechanisms implied that Smo might undergo both types of modification. To determine if Smo could be monoubiquitinated, Myc-Smo or its KR variants was coexpressed with a HA-tagged mutant form of Ub with all Lys residues mutated to Arg (HA-Ub^K0^) in S2 cells. In this case, addition of HA-Ub^K0^ prevents the formation of polyubiquitination chains, generating modified proteins with one or more sites monoubiquitinated. We found that Myc-Smo was effectively modified by HA-Ub^K0^ (Figure 9A). HA-Ub^K0^ was also incorporated into Myc-Smo^K6R^, Myc-Smo^K7R^, and Myc-Smo^K13R^, albeit with reduced efficiency compared with Myc-Smo (Figure 9A), suggesting that Smo can be monoubiquitinated at multiple sites.

In the absence of proteasome inhibitor, HA-Ub^K0^ and wild type HA-Ub were incorporated into Myc-Smo at similar levels (Figure 9B), suggesting that the ubiquitinated Smo species modified by HA-Ub^K0^ or HA-Ub detected under these conditions were mostly mono- or multi-ubiquitinated. Furthermore, Hh stimulation inhibited Smo ubiquitination under these conditions (Figure 9B). However, after MG132 treatment, more HA-Ub conjugated Smo was detected than HA-Ub^K0^ modified Smo (Figure 9B), suggesting that a fraction of Myc-Smo underwent polyubiquitination that was normally degraded by proteasome. The proteasome inhibitor also increased the level of HA-Ub^K0^ conjugated Smo (Figure 9B), suggesting that a fraction of HA-Ub^K0^ conjugated Smo might undergo polyubiquitination via endogenous Ub.

To confirm that Smo could be modified by Lys 48-linked polyubiquitination, we probed Smo immunopurified from S2 cells stably expressing Myc-Smo with a Lys 48-linkage specific polyubiquitin antibody (K48, Cell Signaling). As shown in Figure 9C, immunoprecipitated Myc-Smo was recognized by the K48 antibody and the signal was markedly increased by MG132 treatment, suggesting that Smo can also be modified by Lys 48-linked polyubiquitination that targets it for proteasome-mediated degradation.

## Discussion

Regulation of Smo cell surface expression is a key step in Hh signal transduction [7],[11],[13], but the underlying mechanism has remained unknown. In this study, we provide the first evidence that Smo is ubiquitinated in a manner regulated by Hh signaling and PKA/CK1-mediated Smo phosphorylation. We provide both genetic and biochemical evidence that Smo ubiquitination regulates its endocytic trafficking and cell surface expression. In addition, we provide evidence that the non-visual β-arrestin Krz acts in parallel with Smo ubiquitination to promote its internalization and that Smo ubiquitination is antagonized by the deubiquitinating enzyme UBPY.

Several lines of evidence suggest that the ubiquitin pathway regulates Smo endocytic trafficking and degradation: (1) Smo was accumulated in mutant clones lacking the ubiquitin-activating enzyme Uba1 in wing imaginal discs, and inactivation of Uba1 in S2 cells inhibited Smo ubiquitination and promoted its cell surface accumulation; (2) Smo was accumulated when the activity of several endocytic components or lysosome was inhibited; (3) Hh and PKA/CK1-mediated Smo phosphorylation inhibited Smo ubiquitination and increased Smo cell surface expression; (4) the Smo autoinhibitory domain (SAID) promoted receptor ubiquitination and internalization; (5) Smo was ubiquitinated at multiple sites both inside and outside the SAID domain and mutating the ubiquitin acceptor sites in SAID increased Smo half-life and cell surface expression; and (6) Smo cell surface expression was promoted by the deubiquitinating enzyme UBPY that binds Smo and counteracts Smo ubiquitination.

**Figure 10:**
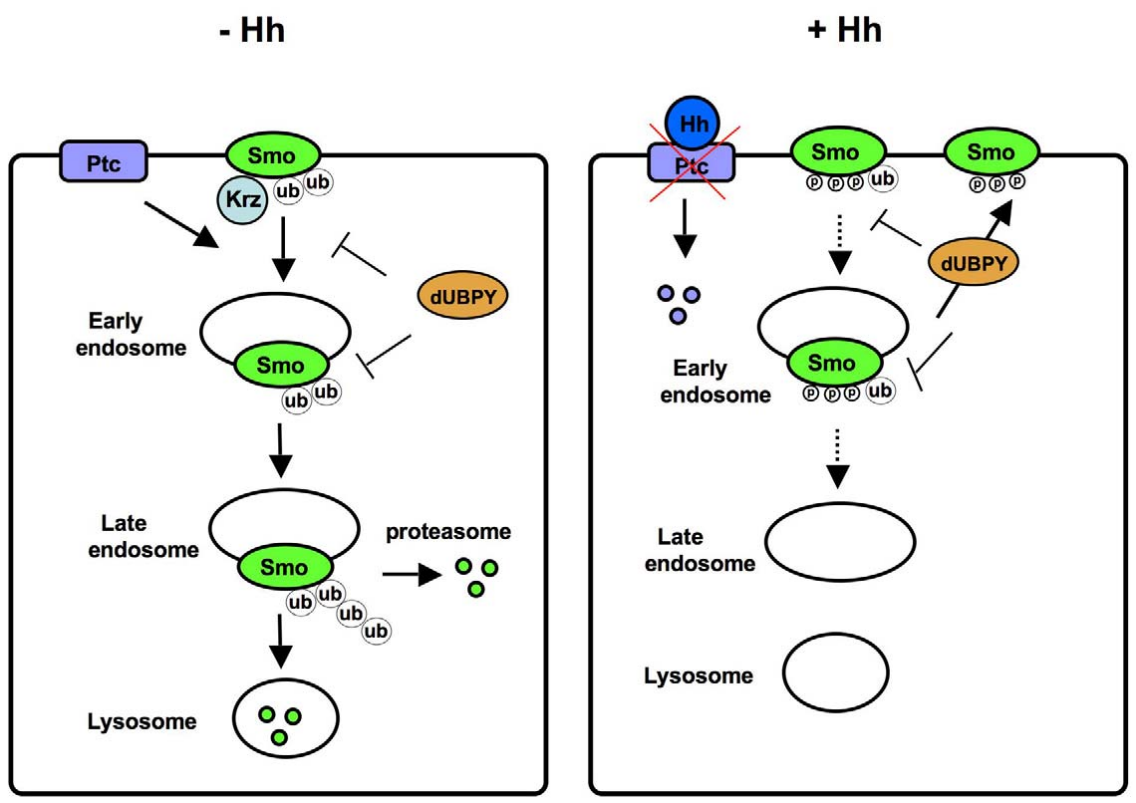
A model for ubiquitin regulation of Smo. In the absence of Hh, Ptc inhibits Smo phosphorylation. Unphosphorylated or under-phosphorylated Smo is effectively ubiquitinated at multiple sites. In addition, Krz binds Smo and acts in parallel with Smo ubiquitination to promote Smo endocytosis. Smo is further ubiquitinated in the endocytic pathway and degraded by both proteasome and lysosome. In the presence of Hh, binding of Hh to Ptc inhibits its activity and promotes its degradation, allowing Smo phosphorylation by PKA and CK1. Phosphorylation inhibits Smo ubiquitination and its association with Krz, thereby inhibiting its internalization. UBPY catalyzes Smo deubiquitination in both signal “off” and “on” states and may facilitate Smo recycling back to the cell surface. See text for details.

Early studies with yeast membrane receptors provided evidence that monoubiquitination of GPCRs mediates their agonist-induced internalization [33],[34]. Later studies with mammalian GPCRs and other receptors suggested that both mono- and polyubiquitination could be involved in receptor endocytosis and degradation [18]. However, it has been shown that “polyubiquitination” of some receptors is due to monoubiquitination at multiple sites (multiubiquitination) instead of forming a polyubiquitination chain at a single site [35],[36]. Here we provide evidence that Smo is both mono- and polyubiquitinated. It is possible that mono- or multiubiquitination may lead to Smo internalization and that internalized Smo could be further ubiquitinated in the endocytic pathway, leading to the formation of Lys 48-linked polyubiquitin chain that targets Smo for proteasome-mediated degradation (Figure 10). Thus, multiple ubiquitination events provide a robust mechanism for Smo downregulation to prevent aberrant Smo activity in the absence of Hh.

Regulation of Smo trafficking and cell surface expression provides a new paradigm for how the ubiquitin pathway controls the activity of a membrane receptor. Unlike all the other cases whereby receptor ubiquitination is triggered by ligand or agonist stimulation and serves as a mechanism to control the duration of cell signaling, Smo ubiquitination occurs in the absence of ligand stimulation and serves as a mechanism to keep the basal pathway activity in check. Smo ubiquitination is inhibited upon ligand stimulation; as a consequence, Smo is accumulated on the cell surface where it becomes activated. Thus, the regulation of Smo ubiquitination by the upstream signal is in the opposite direction compared with other receptors.

How does Hh block Smo ubiquitination? Smo intracellular regions such as SAID could recruit one or more E3 ubiquitin ligases to catalyze Smo ubiquitination and E3 recruitment could be inhibited by Hh stimulation and PKA/CK1-mediated Smo phosphorylation. An alternative but not mutually exclusive mechanism is that Hh and Smo phosphorylation could promote Smo deubiquitination by regulating the binding and/or activity of one or more DUBs. In a systematic RNAi-based screen, we identified UBPY as a Smo DUB. UBPY binds Smo C-tail and antagonizes Smo ubiquitination. UBPY may modulate Smo cell surface expression by attenuating Smo endocytosis and/or promoting Smo recycling (Figure 10). However, we found that UBPY decreases Smo ubiquitination regardless of the Hh signaling states and that the association between UBPY and Smo is not significantly affected by either Hh stimulation or Smo phosphorylation, suggesting that Smo deubiquitination by UBPY is unlikely to be a major mechanism by which Hh inhibits Smo ubiquitination, although we cannot rule out the possibility that Hh regulates UBPY binding to Smo in a subtle way that escaped the detection by our coimmunoprecipitation assay. The mechanism underlying the regulation of Smo ubiquitination might be analogous to those regulating the phosphorylation of many proteins in which kinases instead of phosphatases are usually regulated by upstream signals. Thus, identifying the E3 ligase(s) involved in Smo ubiquitination may shed important light on the mechanism by which Smo ubiquitination is regulated.

We have also obtained evidence that the non-visual β-arrestin Krz can promote Smo internalization by binding to its C-tail and this activity is inhibited by Hh. However, while Krz overexpression effectively internalized Smo, loss-of-Krz-function did not lead to a significant change in Smo cell surface expression (Figure 7C,G) [31]. Our results suggest that Smo ubiquitination can act independently of Krz to internalize Smo, leading to its degradation by both proteasome and lysosome so that the requirement of Krz in internalizing Smo can only be revealed when Smo ubiquitination is compromised (Figure 7G–I). It is possible that Smo ubiquitination plays a major role while Krz only plays a minor role in the regulation of Smo trafficking and cell surface expression.

The mechanisms that regulate Smo trafficking and cell surface expression exhibit interesting similarities to as well as important differences from those regulating GPCRs. For example, it has been shown that agonist-induced downregulation of β2-Adrenergic Receptor (β2AR) is mediated by both β-arrestin and receptor ubiquitination [27]. In addition, β2AR internalization and degradation is regulated by both proteasome- and lysosome-dependent mechanisms [27],[37]. However, β2AR ubiquitination is induced by agonist and serves as a mechanism for desensitization [27],[37], whereas Smo ubiquitination is inhibited by Hh and serves as a mechanism for keeping pathway activity off in the absence of the ligand. β-arrestin binding to β2AR is induced by agonists and requires GRK2-mediated phosphorylation of the activated receptor [27], whereas Krz binding to Smo is attenuated by Hh and Smo phosphorylation (Figure 7). Although GPRK2/GRK2 also regulates Smo in *Drosophila*, its function appears to be uncoupled from that of Krz because loss of GPRK2 exhibits a phenotype distinct from that exhibited by loss of Krz [31],[38]–[40]. Furthermore, Krz can internalize Smo in the absence of GPRK2 [31]. β-arrestin is required for β2AR ubiquitination [27],[37], whereas Krz inactivation does not significantly affect Smo ubiquitination (unpublished observations). Finally, while the proteasome inhibitor MG132 blocks agonist-induced β2AR internalization [27], it does not prevent Smo internalization but instead inhibits Smo degradation after internalization (Figure 3).

It is also interesting to note that β-arrestin has been implicated in the regulation of Smo trafficking and Shh signaling in vertebrates [29],[41],[42]. Furthermore, β-arrestin binds to mammalian Smo (mSmo) in a manner promoted by Shh and GRK2-mediated phosphorylation of mSmo C-tail [42],[43], which is analogous to agonist-induced β-arrestin binding to GPCRs. However, instead of internalizing mSmo for degradation, β-arrestin appears to promote mSmo ciliary accumulation [42], which correlates with its positive role in Shh signaling. Both *Drosophila* and vertebrate Smo proteins can activate trimeric G-proteins [44]–[46], suggesting that they are not only structurally but also functionally related to GPCRs. It is conceivable that Smo proteins may employ multiple mechanisms utilized by GPCRs to control their intracellular trafficking and activity. Thus, it will be interesting to determine whether vertebrate Smo is also regulated by the ubiquitin pathway.

## Materials and Methods

### Mutations and Transgenes

Mutations used in this study are *Uba1^H33^* [19], *l(2)23Ad^D28^*/*hrs* [23], *krz^1^* [47], and *UBPY^KO^* [48]. Mutant clones were generated by *FLP/FRT*-mediated mitotic recombination as previously described [49]. The genotypes for making clones are as follows: *Uba1* clones: *yw 122; FRT42 Uba1^H33^ /FRT42 hs-Myc-GFP*; hrs clones: *yw 122; l(2)23Ad^D28^ FRT40/ hs-Myc-GFP FRT40*; *krz* or *UBPY* clones: *yw 122; FRT82 krz^1^ or UBPY^KO^ /FRT82 hs-Myc-GFP*. Transgenic RNAi lines used are *UAS-Tsg101-RNAi* (VDRC# 23944), *UAS-Avl-RNAi* (VDRC# 5413), and *UAS-Rab5-RNAi* (VDRC# 34096). UAS-Krz and UAS-UBPY are previously described [47],[48]. Constructs for various tagged forms of wild type Smo, Smo^ΔCT^, Smo^CT^, Smo^Δ661–818^, Smo^SA^, and Smo^SD^ are previously described [8],[13],[50]. CFP-tagged Fz2 is described [8]. To construct Fz2/Smo chimeric proteins, the coding sequence for the wild type and mutant forms of SAID (aa 661–818) was amplified by PCR and inserted at a Kpn I site between the coding sequence for Fz2 and CFP. To construct Krz-YFP, the coding sequence of Krz was amplified by PCR and inserted between Not I/ Kpn I digestion sites of *pUAST* vector, and YFP was inserted in frame to the C-terminus of Krz between Kpn I/ XbaI digestion sites. Smo^K6R^, Smo^K7R^, and Smo^K13R^ were generated using PCR-based site-directed mutagenesis to introduce K to R mutations in corresponding Lys residues.

### Cell Culture, Transfection, Immunoprecipitation, Western Blot, and Immunostaining

*Drosophila* S2 cells were cultured in *Drosophila* SFM (Invitrogen) with 10% fetal bovine serum, 100 U/ml of penicillin, and 100 mg/ml of streptomycin at 23°C. Transfection was carried out by Calcium Phosphate Transfection Kit (Specialty Media) according to the manufacturer's instructions. Hh-conditioned medium treatment was carried out as described [51]. Cells were treated with 50 µM MG132 (Calbiochem) for 4 h to inhibit proteasome or 20 mM NH_4_Cl (Sigma) for 18 h to inhibit lysosome. Immunoprecipitation and Western blot analysis were carried out using standard protocols as previously described [52]. For Smo cell surface staining assay, S2 cells were harvested and washed with PBS, fixed with 4% formaldehyde at room temperature for 20 min, and incubated with the mouse anti-SmoN antibody in PBS at room temperature for 90 min. Cells were washed 3 times by PBS followed by secondary antibody staining. Immunostaining of imaginal discs was carried out as described [13],[49]. Quantification of immunostaining and autoradiography densitometric analysis was performed using ImageJ software. Antibodies used in this study were: mouse anti-SmoN (DSHB), rat anti-Ci 2A1 [53], rabbit and mouse anti-Flag (Sigma), mouse anti-Myc (Santa Cruz), mouse anti-HA (Santa Cruz), mouse anti-GFP (Millipore), rabbit anti-GFP (Santa Cruz), rabbit anti-LacZ (ICN Pharmaceuticals, Inc.), anti-Ub (P4D1) (Santa Cruz), and anti-Poly-Ub^K48^ (Cell signaling).

### Ubiquitination Assay

Ubiquitination assays were carried out based on the protocol described previously [21]. Briefly, Myc-Smo stably expressing S2 cells or S2 cells transfected with Smo variants with or without HA-Ub (wild type or mutants) were treated with MG132 or NH_4_Cl before harvesting. Cells were lysed in 100 µl of denaturing buffer (1% SDS/50 mM Tris, pH 7.5/0.5 mM EDTA/1 mM DTT). After incubation for 5 min at 100°C, the lysates were diluted 10-fold with lysis buffer and then subjected to immunoprecipitation and Western blot analysis.

### RNAi in *Drosophila* S2 Cells

dsRNA was generated by MEGAscript High Yield Transcription Kit (Ambion: #AM1334) according to the manufacturer's instruction. DNA templates targeting Uba1(aa 1–172), Krz(aa 191–365), UBPY(aa 25–191 and aa 124–290) or other DBUs (Table S1) were generated by PCR and used for generating dsRNA. Ptc RNAi was carried out as previously described [51]. dsRNA targeting the Fire Fly Luciferase coding sequence was used as a control. For RNAi knockdown experiments, S2 cells were cultured in serum free medium containing indicated dsRNA at 23°C for 8 h. After adding fetal bovine serum to a final concentration of 10%, dsRNA treated cells were cultured overnight before transfection. 48 h after transfection, cells were harvested for further analysis.

## Supporting Information

Figure S1 Smo is accumulated on the cell surface in *Uba1* mutant clones. Low (A, B) and high (A', B') magnification view of wing imaginal discs carrying *Uba1^H33^* mutant clones and immunostained with anti-SmoN (red) and anti-GFP (green) antibodies. Larvae were grown at 18°C after clone induction and shifted to 30°C for 24 (A, A') or 12 (B, B') h, followed by immunostaining with anti-SmoN antibody prior to membrane permeabilization. *Uba1^H33^* mutant clones are marked by the lack of GFP staining. Arrows indicate anterior clones that accumulate Smo on the cell surface.
(TIF)

Figure S2 Smo^K13R^ is more stable than wild type Smo in vivo. Wing discs expressing *UAS-Myc-Smo* (left) or *UAS-Myc-Smo^K13R^* under the control of *MS1096* in conjunction with *Gal80^ts^*. Larvae were grown at 18°C until late third instar, shifted to 30°C for 12 h, and then put back to 18°C for the indicated hours before immunostaining with anti-Myc antibody. Arrows indicate Myc-Smo or Myc-Smo^K13R^ accumulation in anterior compartment cells distant from the A/P boundary.
(TIF)

Figure S3 Krz acts in parallel with ubiquitination to internalize Smo. Myc-Smo expressing S2 cells were treated with Luc, Uba1, or Uba1 plus Krz dsRNA in the absence or presence of PYR41, followed by immunostaining to visualize cell surface Smo or total Smo. Quantification of cell surface and total Smo levels was shown (20 cells for each condition). The numbers indicate the ratio of cell surface Smo signal versus total Smo signal.
(TIF)

Figure S4 An RNAi screen identified UBPY as a Smo DUB. S2 cells stably expressing Myc-Smo were treated with control dsRNA or dsRNA targeting the indicated DUB. After treatment with MG132, cell extracts were immunoprecipitated with anti-Myc antibody, followed by immunoblotting with anti-Myc or anti-Ub antibody. Loading was normalized by the amount of Myc-Smo monomer. IP, immunoprecipitation; IB, immunoblot.
(TIF)

Table S1 Annotated DUBs in the *Drosophila* genome. A list of annotated *Drosophila* DUBs with gene names, CG numbers, and primer sequences for making dsRNA are indicated. The dsRNAs against individual DUBs are designed based on the sequence and primer information through the Gene and Reagent Lookup tool on the DRSC website: http://www.flyrnai.org/cgi-bin/RNAi_gene_lookup_public.pl.
(DOC)

## Abbreviations

A: anterior
Avl: Avalanche
β2AR: β2-Adrenergic Receptor
CHX: cycloheximide
Ci: Cubitus interruptus
C-tail: carboxyl intracellular tail
*dpp*: *decapentaplegic*
dsRNA: double-stranded RNA
FS: Fz2-SAID
Fz2: Frizzle 2
GPCR: G protein coupled receptor
Hh: Hedgehog
Hrs: HGF-regulated tyrosine kinase substrate
Krz: Kurtz
mSmo: mammalian Smo
MVBs: multivesicular bodies
P: posterior
Ptc: Patched
RTK: receptor tyrosine kinase
SAID: Smo autoinhibitory domain
Smo: Smoothened
Wg: Wingless
