# Supplementary figures and images for: Hedgehog-Regulated Ubiquitination Controls Smoothened Trafficking and Cell Surface Expression in Drosophila
Source: PLoS Biol. 2012 Jan 10;10(1):e1001239. doi: 10.1371/journal.pbio.1001239 (PMC3254653; doi:10.1371/journal.pbio.1001239)

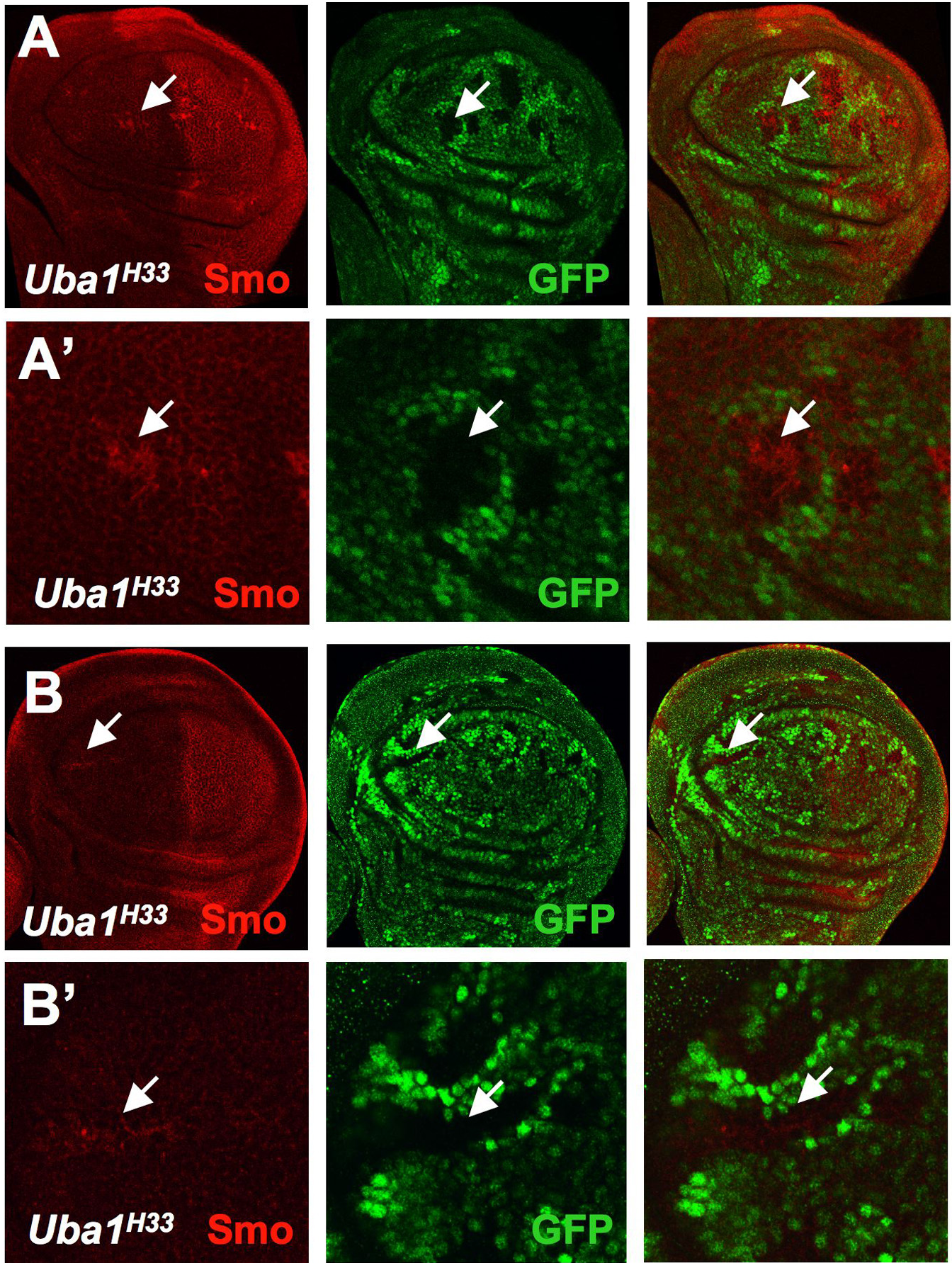

Supplement: Figure S1 — Smo is accumulated on the cell surface in Uba1 mutant clones. Low (A, B) and high (A', B') magnification view of wing imaginal discs carrying Uba1H33 mutant clones and immunostained with anti-SmoN (red) and anti-GFP (green) antibodies. Larvae were grown at 18°C after clone induction and shifted to 30°C for 24 (A, A') or 12 (B, B') h, followed by immunostaining with anti-SmoN antibody prior to membrane permeabilization. Uba1H33 mutant clones are marked by the lack of GFP staining. Arrows indicate anterior clones that accumulate Smo on the cell surface. (TIF) [file pbio.1001239.s001.tif]

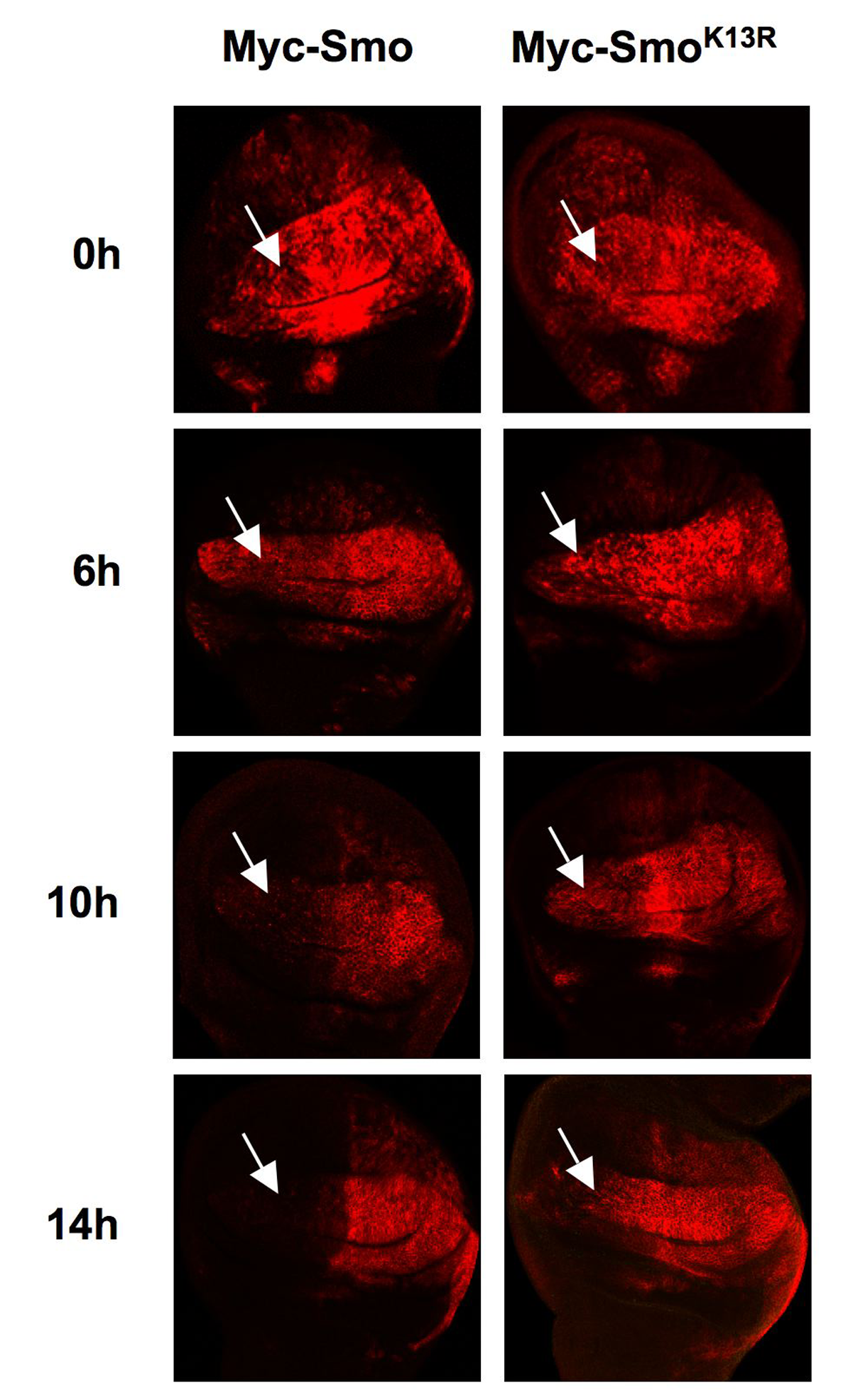

Supplement: Figure S2 — SmoK13R is more stable than wild type Smo in vivo. Wing discs expressing UAS-Myc-Smo (left) or UAS-Myc-SmoK13R under the control of MS1096 in conjunction with Gal80ts. Larvae were grown at 18°C until late third instar, shifted to 30°C for 12 h, and then put back to 18°C for the indicated hours before immunostaining with anti-Myc antibody. Arrows indicate Myc-Smo or Myc-SmoK13R accumulation in anterior compartment cells distant from the A/P boundary. (TIF) [file pbio.1001239.s002.tif]

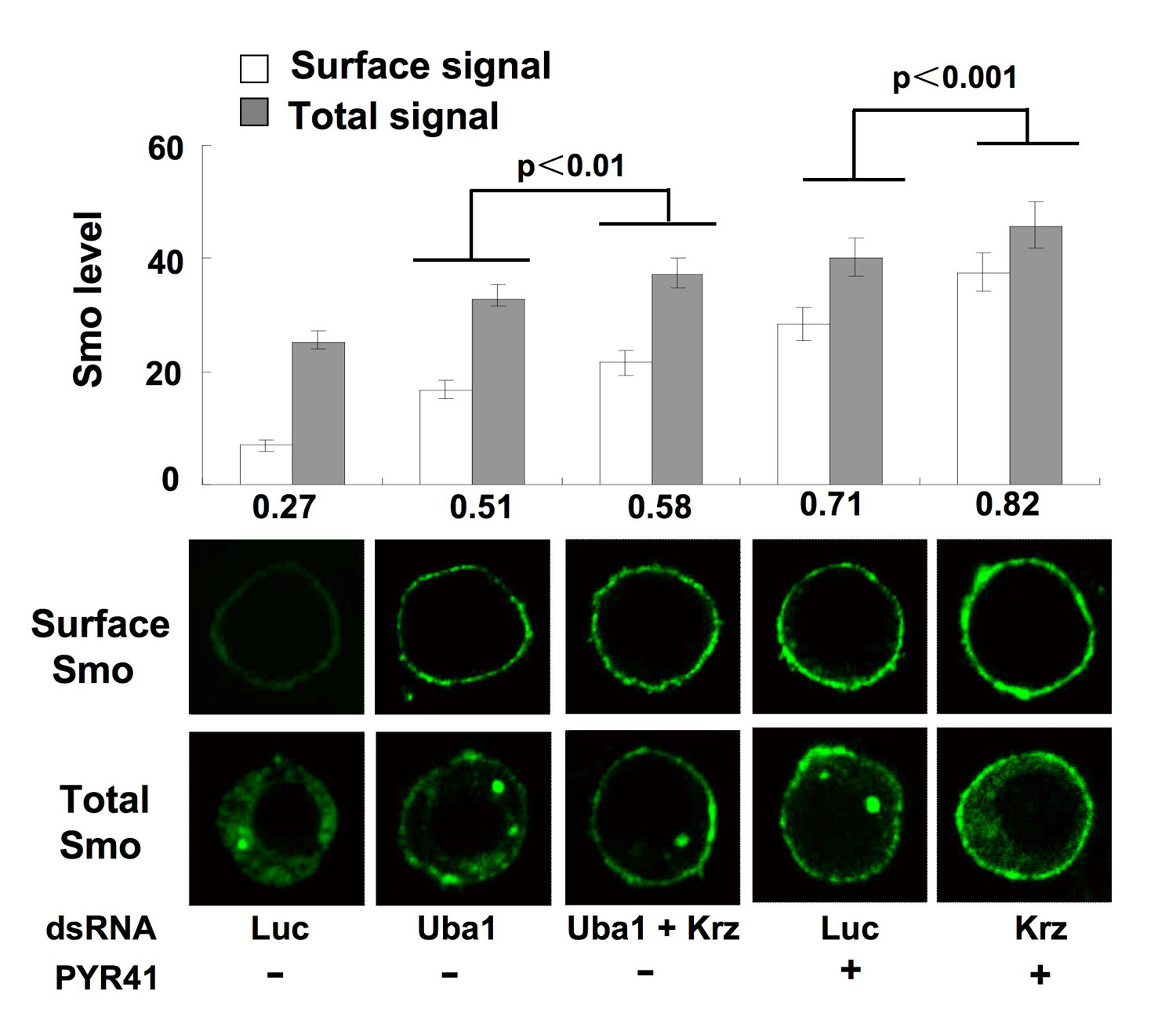

Supplement: Figure S3 — Krz acts in parallel with ubiquitination to internalize Smo. Myc-Smo expressing S2 cells were treated with Luc, Uba1, or Uba1 plus Krz dsRNA in the absence or presence of PYR41, followed by immunostaining to visualize cell surface Smo or total Smo. Quantification of cell surface and total Smo levels was shown (20 cells for each condition). The numbers indicate the ratio of cell surface Smo signal versus total Smo signal. (TIF) [file pbio.1001239.s003.tif]

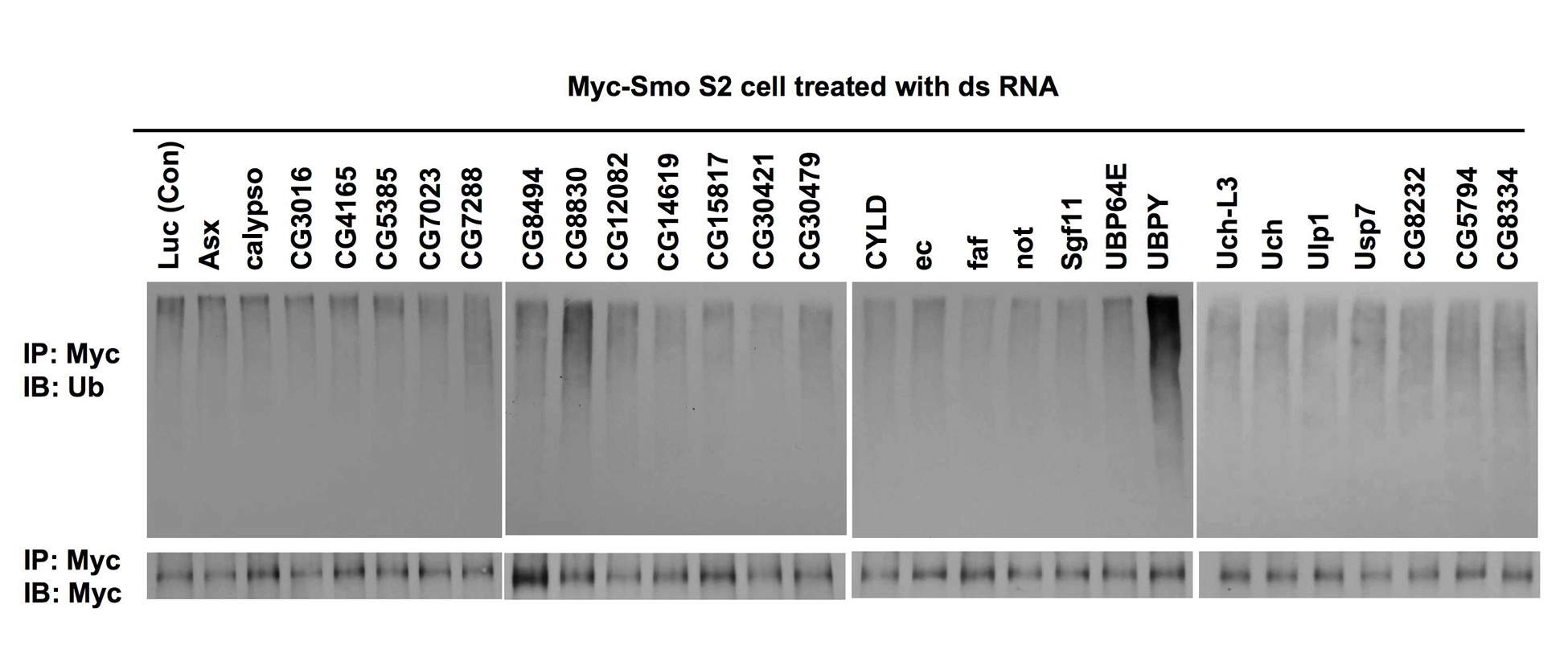

Supplement: Figure S4 — An RNAi screen identified UBPY as a Smo DUB. S2 cells stably expressing Myc-Smo were treated with control dsRNA or dsRNA targeting the indicated DUB. After treatment with MG132, cell extracts were immunoprecipitated with anti-Myc antibody, followed by immunoblotting with anti-Myc or anti-Ub antibody. Loading was normalized by the amount of Myc-Smo monomer. IP, immunoprecipitation; IB, immunoblot. (TIF) [file pbio.1001239.s004.tif]
