# Supplementary material for: Hedgehog-Regulated Ubiquitination Controls Smoothened Trafficking and Cell Surface Expression in Drosophila
Source: PLoS Biol. 2012 Jan 10;10(1):e1001239. doi: 10.1371/journal.pbio.1001239 (PMC3254653; doi:10.1371/journal.pbio.1001239)
Supplement: Table S1 — Annotated DUBs in the Drosophila genome. A list of annotated Drosophila DUBs with gene names, CG numbers, and primer sequences for making dsRNA are indicated. The dsRNAs against individual DUBs are designed based on the sequence and primer information through the Gene and Reagent Lookup tool on the DRSC website: http://www.flyrnai.org/cgi-bin/RNAi_gene_lookup_public.pl. (DOC) [file pbio.1001239.s005.doc]

**Table S1**

| Name | CG No. | 5' primer | 3' primer |
| --- | --- | --- | --- |
| Asx | CG8787 | TAATACGACTCACTATAGGGCGCAGATTGAGCAAACAA | TAATACGACTCACTATAGGGCCCTAGAATTCTTCTCGC |
| [calypso](http://flybase.org/cgi-bin/fbidq.html?FBgn0262166) | [CG8445](http://flybase.org/cgi-bin/gbrowse/dmel/?dontadjust=1&name=FBgn0262166) | TAATACGACTCACTATAGGGGTGTATCCGGACGTTGTG | TAATACGACTCACTATAGGGCACCCTGAGTCGACTAAAA |
| [CG3016](http://flybase.org/cgi-bin/fbidq.html?FBgn0029819) | [CG3016](http://flybase.org/cgi-bin/gbrowse/dmel/?dontadjust=1&name=FBgn0029819) | TAATACGACTCACTATAGGGTGCAAACTGGTGCTCTCA | TAATACGACTCACTATAGGGTGCTTCCTAAACACCCTGC |
| [CG4165](http://flybase.org/cgi-bin/fbidq.html?FBgn0029763) | [CG4165](http://flybase.org/cgi-bin/gbrowse/dmel/?dontadjust=1&name=FBgn0029763) | TAATACGACTCACTATAGGGCAAGTCGGTGAACACCAA | TAATACGACTCACTATAGGGAGTTGGTCATCGTCCTGG |
| [CG5384](http://flybase.org/cgi-bin/fbidq.html?FBgn0032216) | [CG5384](http://flybase.org/cgi-bin/gbrowse/dmel/?dontadjust=1&name=FBgn0032216) | TAATACGACTCACTATAGGGATTCGAGGGTTCCTGGTT | TAATACGACTCACTATAGGGCGTGCTACATGAATGCCA |
| [CG7023](http://flybase.org/cgi-bin/fbidq.html?FBgn0039025) | [CG7023](http://flybase.org/cgi-bin/gbrowse/dmel/?dontadjust=1&name=FBgn0039025) | TAATACGACTCACTATAGGGAAGGGTGTTTTGCGACTC | TAATACGACTCACTATAGGGACTCCTCCTTCTCCTTTC |
| [CG7288](http://flybase.org/cgi-bin/fbidq.html?FBgn0030969) | [CG7288](http://flybase.org/cgi-bin/gbrowse/dmel/?dontadjust=1&name=FBgn0030969) | TAATACGACTCACTATAGGGCCTGCCGGATAATTACGA | TAATACGACTCACTATAGGGTTCCACATCTTGCGCATTA |
| [CG8494](http://flybase.org/cgi-bin/fbidq.html?FBgn0033916) | [CG8494](http://flybase.org/cgi-bin/gbrowse/dmel/?dontadjust=1&name=FBgn0033916) | TAATACGACTCACTATAGGGTCCGCTGGAGGGCTTCGA | TAATACGACTCACTATAGGGTCCGCCACCGAAAGTCCG |
| [CG8830](http://flybase.org/cgi-bin/fbidq.html?FBgn0033738) | [CG8830](http://flybase.org/cgi-bin/gbrowse/dmel/?dontadjust=1&name=FBgn0033738) | TAATACGACTCACTATAGGGCATCGATAGGTGGTGGAA | TAATACGACTCACTATAGGGTGCTTATCGAGTGCAATTCA |
| [CG12082](http://flybase.org/cgi-bin/fbidq.html?FBgn0035402) | [CG12082](http://flybase.org/cgi-bin/gbrowse/dmel/?dontadjust=1&name=FBgn0035402) | TAATACGACTCACTATAGGGGGCAGCTTCTTATCCAGG | TAATACGACTCACTATAGGGCCTCCCATCTACAAGGAC |
| [CG14619](http://flybase.org/cgi-bin/fbidq.html?FBgn0031187) | [CG14619](http://flybase.org/cgi-bin/gbrowse/dmel/?dontadjust=1&name=FBgn0031187) | TAATACGACTCACTATAGGGTGCATTACTCGCTCTATG | TAATACGACTCACTATAGGGCATCCTACCCCATGGTCA |
| [CG15817](http://flybase.org/cgi-bin/fbidq.html?FBgn0028476) | [CG15817](http://flybase.org/cgi-bin/gbrowse/dmel/?dontadjust=1&name=FBgn0028476) | TAATACGACTCACTATAGGGTACATAGCCAACCCGGAT | TAATACGACTCACTATAGGGCACAATGCCTTCGAAGTC |
| [CG30421](http://flybase.org/cgi-bin/fbidq.html?FBgn0050421) | [CG30421](http://flybase.org/cgi-bin/gbrowse/dmel/?dontadjust=1&name=FBgn0050421) | TAATACGACTCACTATAGGGCACATTCGACCCCTTTCA | TAATACGACTCACTATAGGGTATGAGCAGTCACGAGGT |
| [CG32479](http://flybase.org/cgi-bin/fbidq.html?FBgn0052479) | [CG32479](http://flybase.org/cgi-bin/gbrowse/dmel/?dontadjust=1&name=FBgn0052479) | TAATACGACTCACTATAGGGCCAGTTCATCAGCCAATC | TAATACGACTCACTATAGGGCTTGTGGCGCGTCAGGTA |
| [CYLD](http://flybase.org/cgi-bin/fbidq.html?FBgn0032210) | [CG5603](http://flybase.org/cgi-bin/gbrowse/dmel/?dontadjust=1&name=FBgn0032210) | TAATACGACTCACTATAGGGTCAATTATGTCTGTGACA | TAATACGACTCACTATAGGGTCGGGGCAAGATTCTTAT |
| [ec](http://flybase.org/cgi-bin/fbidq.html?FBgn0000542) | [CG2904](http://flybase.org/cgi-bin/gbrowse/dmel/?dontadjust=1&name=FBgn0000542) | TAATACGACTCACTATAGGGACAGTGGACGCAGAATGA | TAATACGACTCACTATAGGGGTACGGCACAATTGAGAAA |
| [faf](http://flybase.org/cgi-bin/fbidq.html?FBgn0005632) | [CG1945](http://flybase.org/cgi-bin/gbrowse/dmel/?dontadjust=1&name=FBgn0005632) | TAATACGACTCACTATAGGGAAGACACTAACGTGGGCG | TAATACGACTCACTATAGGGCCCTCTTCTGGTAGTGCG |
| not | [CG4166](http://flybase.org/cgi-bin/gbrowse/dmel/?dontadjust=1&name=FBgn0013717) | TAATACGACTCACTATAGGGCGCAGGCTGAACTGTTTG | TAATACGACTCACTATAGGGTCTATTCCGGCTCCCGTT |
| [Sgf11](http://flybase.org/cgi-bin/fbidq.html?FBgn0036804) | [CG13379](http://flybase.org/cgi-bin/gbrowse/dmel/?dontadjust=1&name=FBgn0036804) | TAATACGACTCACTATAGGGGTTGTTCCTTGAGTTCTG | TAATACGACTCACTATGGGAAACCAAGTTCCCACAACT |
| [Ubp64E](http://flybase.org/cgi-bin/fbidq.html?FBgn0016756) | [CG5486](http://flybase.org/cgi-bin/gbrowse/dmel/?dontadjust=1&name=FBgn0016756) | TAATACGACTCACTATAGGGTCAGTTCGGAATCAAGTC | TAATACGACTCACTATAGGGGTCGGACTAGCAGATGCT |
| [Ubpy](http://flybase.org/cgi-bin/fbidq.html?FBgn0038862) | [CG5798](http://flybase.org/cgi-bin/gbrowse/dmel/?dontadjust=1&name=FBgn0038862) | TAATACGACTCACTATAGGGCAGAACGATGAGCAGTTG | TAATACGACTCACTATAGGGCCTCGGCTGCGGTTTCAT |
| [Uch-L3](http://flybase.org/cgi-bin/fbidq.html?FBgn0011327) | [CG3431](http://flybase.org/cgi-bin/gbrowse/dmel/?dontadjust=1&name=FBgn0011327) | TAATACGACTCACTATAGGGCTGCATGCGCTTCTCAAT | TAATACGACTCACTATAGGGGAGCAACGCTAGCCAGATT |
| [Uch](http://flybase.org/cgi-bin/fbidq.html?FBgn0010288) | [CG4265](http://flybase.org/cgi-bin/gbrowse/dmel/?dontadjust=1&name=FBgn0010288) | TAATACGACTCACTATAGGGCGCGGTCAAGGCCAAAAC | TAATACGACTCACTATAGGGACATTGACCGCGGAGTAC |
| [Ulp1](http://flybase.org/cgi-bin/fbidq.html?FBgn0027603) | [CG12359](http://flybase.org/cgi-bin/gbrowse/dmel/?dontadjust=1&name=FBgn0027603) | TAATACGACTCACTATAGGGATTTCTAAGCCCATCGAA | TAATACGACTCACTATAGGGTTGGAAATATCCCAGGAA |
| [Usp7](http://flybase.org/cgi-bin/fbidq.html?FBgn0030366) | [CG1490](http://flybase.org/cgi-bin/gbrowse/dmel/?dontadjust=1&name=FBgn0030366) | TAATACGACTCACTATAGGGACATAAACTGGCGGCGAT | TAATACGACTCACTATAGGGGCATCCAGTGAGTAGCGA |
| CG8232 | CG8232 | TAATACGACTCACTATAGGGCATGCTACCGCACTTGAC | TAATACGACTCACTATAGGGCTTTTATCCAGCATGTTG |
| CG5794 | CG5794 | TAATACGACTCACTATAGGGAAAGCCAGTACGCAAGTG | TAATACGACTCACTATAGGGCGTCAGAAGAAAGGCGAA |
| CG8334 | CG8334 | TAATACGACTCACTATAGGGGCTGTTTAATACCCAACC | TAATACGACTCACTATAGGGAGACCTCAAAGTAGATAT |
